# Persistent Homology Classifies Parameter Dependence of Patterns in Turing Systems

**DOI:** 10.1007/s11538-025-01552-9

**Published:** 2025-12-24

**Authors:** Reemon Spector, Heather A. Harrington, Eamonn A. Gaffney

**Affiliations:** 1 Mathematical Institute, University of Oxford, England, United Kingdom; 2 Division of Infection and Immunity, University College London, London, United Kingdom; 3 Max Planck Institute of Molecular Cell Biology and Genetics, Dresden, Germany; 4 Centre for Systems Biology, Dresden, Germany; 5 Faculty of Mathematics, Technische Universität Dresden, Dresden, Germany

## Abstract

This paper illustrates a further application of topological data analysis to the study of self-organising models for chemical and biological systems. In particular, we investigate whether topological summaries can capture the parameter dependence of pattern topology in reaction diffusion systems, by examining the homology of sublevel sets of solutions to Turing reaction diffusion systems for a range of parameters. We demonstrate that a topological clustering algorithm can reveal how pattern topology depends on parameters, using the chlorite–iodide–malonic acid system, and the prototypical Schnakenberg system for illustration. In addition, we discuss the prospective application of such clustering, for instance in refining priors for detailed parameter estimation for self-organising systems.

## Introduction

Turing’s seminal work on the theory of morphogenesis introduced the diffusion-driven instability, where two interacting molecules or species, often referred to as morphogens in biological contexts, exhibit a stable steady state in the absence of diffusive transport, but destabilise to generate spatially heterogeneous patterns once diffusion becomes significant (Turing 1952). In particular, this entails that spontaneous self-organisation can emerge from essentially homogeneous systems via a supercritical bifurcation that is often driven by physically simple bifurcation parameters, such as domain size (Gierer and Meinhardt 1972; Murray 2003). This has been eponymously labelled as the Turing mechanism and may drive the emergence of patterns not only in developmental biology as originally suggested by Turing (Turing 1952), for instance with Nodal-Lefty interactions (Müller et al. 2012) and mammalian palate ridges (Economou et al. 2012), but also in a vast array of systems more generally. The latter encompass, inter alia, mollusc shell decoration (Meinhardt and Klingler 1987), chemical reaction patterning (Castets et al. 1990) and large-scale vegetation structure (Ge 2023), with a myriad of further applications detailed in a recent editorial celebrating this aspect of Turing’s work and its 70-year legacy (The editors 2022).

While a tremendous effort has been made in recent decades to further our understanding of the Turing mechanism, in particular its limitations and generalisations, multiple challenges remain, for instance the problems of model selection and parameter estimation (Krause et al. 2021; Woolley et al. 2021). In particular, despite the simplicity of the Turing mechanism, whose initiation is well described by linear theory, the long-time behaviours of reaction diffusion models that exhibit Turing patterns post-bifurcation are governed by highly complex and ill-understood nonlinear dynamics. This is especially true for the spatial topology of long-time solutions to reaction diffusion equations, though it is noteworthy that an inherent, and very simple, relation between pattern topology and model parameters was determined over 30 years ago by Ermentrout via a weakly non-linear bifurcation analysis, albeit with the severe restriction of a sufficiently small square domain (Ermentrout 1991). A recent survey underscores the need for more quantitative methods for qualitative data, such as patterns, and highlights techniques from topological data analysis (Volkening 2024).

The field of topological data analysis, used to quantify and classify the shape of data, has developed extensively in recent years (Carlsson 2009; Ghrist 2014; Edelsbrunner and Harer 2010). Persistent homology is one of the prominent tools in topological data analysis, which describes ‘shape’ by computing topological features (i.e., homology) across multiple scales. Informally, the (simplicial) homology of a subset will recover the main topological features of interest: namely the number of connected components, and the number of loops and voids up to continuous deformation. The topological summary of persistent homology is a barcode, which is a multiset of intervals, where each interval or bar represents a topological feature and the endpoints give the scale at which that feature appears and disappears. This metric-dependent topological framework provides an interpretable quantification of features of interest, such as patterns with clusters and holes, at different scales, allowing concrete comparisons of these quantities, and transforming the way data can be used for statistics and machine learning (Otter et al. 2017; Wasserman 2018; Ali et al. 2023). Even initial applications in pattern forming systems span a multitude of areas of research, including the analysis of biological aggregation models (Topaz et al. 2015), spatial structures predicted in angiogenic network simulations (Nardini et al. 2021) and experiments (Stolz et al. 2022), coral resilience models (McDonald 2023) and agent-based frameworks for zebrafish stripe formation (McGuirl et al. 2020) and tumour microenvironment (Yang et al. 2023; Stolz et al. 2024).

In light of these recent methodological developments and the possibility of relatively simple relations between pattern shape on the one hand, and model parameters for Turing systems on the other given Ermentrout’s observations (Ermentrout 1991), the primary goal of this paper is to investigate the prospect of leveraging topological data analysis to classify the topology of select Turing system solutions in the fully nonlinear regime. A secondary objective will be to discuss whether such classification studies are sufficiently informative to facilitate a further understanding of Turing systems.

To facilitate this initial study, we restrict ourselves to systems where the reaction kinetics are considered to be known, to eliminate confounding difficulties from model uncertainty, noting that model misspecification has at least the potential to generate significant impact on predictions even before transport is considered, particularly when dynamical system structural instability is not guaranteed (Kuznetsov 2004). This restriction lends the current study to chemical systems, where there is often much greater certainty in the choice of kinetics rather than biological ones, even in experimentally informed biological modelling (e.g. (Glover et al. 2023)). In contrast, for chemical system patterning, such as that associated with the chlorite–iodide–malonic acid (CIMA) reaction, and the ferrocyanide-iodate-sulfite reaction, the kinetics are relatively well-understood, and we thus choose the CIMA reaction as the prototype exemplar to demonstrate our results (Castets et al. 1990; Lengyel and Epstein 1992; Gaspar and Showalter 1990). As a second model choice, we consider Schnakenberg kinetics, whose nonlinearity can be interpreted in terms of a simple autocatalytic chemical reactions (Schnakenberg 1979). The difference in behaviour between these two models is very distinct, at least sufficiently close to the bifurcation to pattern from the homogeneous steady state, with CIMA an example of pure kinetics and Schnakenberg an example of cross kinetics, spanning the pure-cross dichotomous divide of two-variable reaction diffusion Turing systems (Dillon et al. 1994), ensuring that the two exemplars explored in this study do not have identical behaviours in general.

To proceed in exploring our objectives, Section 2 first recapitulates some of the classical theory for the Turing mechanism and its limitations to motivate the use of persistent homology. We then introduce some of the machinery algebraic topology has to offer in Section 3, and later apply this machinery to data from the solution manifolds of the CIMA and Schnakenberg systems. Having obtained these topological summaries in the form of barcodes at multiple points in the Turing space, we discuss the algorithm used to cluster the emergent patterns in Section 4, and describe some applications of these results to parameter estimation and model selection. In Section 5, we demonstrate that barcodes are sufficient to classify these patterns in the examples we consider, and we turn to discussing these results and assessing their limitations in Section 6, where we also discuss some avenues for further research.

## Background on Turing Patterns

We begin by introducing some necessary background about the chlorite–iodide–malonic acid (CIMA) reaction, a system of reactants that experimentally exhibits Turing patterns (Castets et al. 1990).

Let *X* and *Y* denote iodide ions ($\mathrm {I^-}$) and chlorite ions ($\mathrm {ClO_2^-}$), respectively. In the experimental setup of Castets et al. (1990), the reactants are placed in a chemically inert polyacrylamide gel rich in an immobile starch, *S*, which rapidly and reversibly reacts with *X* to form an essentially immobile complex *SX* (with a very small diffusion coefficient, due to its high molecular weight). Assuming a large excess of *S* that is uniformly distributed in the domain (so that the concentration of *S* is approximately its initial concentration $s_0$ throughout), and that both the formation and the dissociation of the complex are rapid, we obtain that, after a fast transient, the densities of *SX* and *X* will be in quasi-equilibrium (Lengyel and Epstein 1992).

To model this, we closely follow (Lengyel and Epstein 1992, 1991) and begin with the known (reduced) chemical reactions1where *A* is a $\textrm{I}_2$ molecule and *P* is a product which, up to rescaling, forms at an empirically measured nonlinear rate $k_3' \propto \frac{X Y}{w + X^2}$, where *w* is a constant. Assuming *A* is abundant and held uniformly constant, using the quasi-equilibrium assumption, and denoting the densities of *X*, *Y* and *SX* by *x*, *y* and *sx* respectively, Lengyel and Epstein (1992) obtain the relation $\frac{\partial \left( x + sx\right) }{\partial \tau } = \left( 1 + \frac{k_+' s_0}{k_-'}\right) \frac{\partial x}{\partial \tau }$. This yields the equations

2 $$\begin{aligned} \begin{aligned} \left( 1 + \frac{k_+' s_0}{k_-'}\right) \frac{\partial x}{\partial t}&= D_X \nabla ^2 x + k_1' - k_2' x - \frac{4 k_3' x y}{w + x^2}, \\ \frac{\partial y}{\partial t}&= D_Y \nabla ^2 x + k_2' x - \frac{k_3' x y}{w + x^2}, \end{aligned}\end{aligned}$$

where $D_X$ and $D_Y$ represent the diffusivities of *X* and *Y* respectively. After nondimensionalisation via

$$\begin{aligned} t = \left( 1 + \frac{k_+' s_0}{k_-'}\right) \frac{1}{k_2'} \tau ,\ \ z = \frac{\sqrt{D_X}}{\sqrt{k_2'}} \xi , \ \ x(z,t) = \sqrt{w} u(\xi ,\tau ),\ \ y(z,t) = \frac{k_2' w}{k_3'} v(\xi ,\tau ), \end{aligned}$$

we obtain

CIMA $$\begin{aligned} \begin{aligned} \frac{\partial u}{\partial \tau }&= \nabla ^2u + \alpha - u - \frac{4 u v}{1 + u^2}, \\ \frac{\partial v}{\partial \tau }&= \sigma \left( \delta \nabla ^2v + \beta u - \frac{\beta u v}{1 + u^2}\right) , \end{aligned} \end{aligned}$$

where $\alpha , \beta , \delta , \sigma$ are positive constants given by

$$\begin{aligned} \alpha = \frac{k_1'}{k_2' \sqrt{w}},\ \ ~~~\beta = \frac{k_3'}{k_2' \sqrt{w}},\ \ ~~~\delta = \frac{D_Y}{D_X},\ \ ~~~\sigma = 1 + \frac{k_+' s_0}{k_-'}. \end{aligned}$$

For a well-posed, closed system, we impose initial conditions given by $u(\xi ,0) = u_0(\xi )$ and $v(\xi ,0) = v_0(\xi )$, and require (CIMA) to hold on a domain $\Omega \subset \mathbb {R}^2$ with Neumann boundary conditions.

### The Turing Space for the CIMA System

Closely following (Murray 2003, Chapter 2), we compute the *Turing conditions* for the CIMA system, which are the necessary conditions for pattern formation via a diffusion-driven instability. Linearising at the spatially uniform steady state $(u_s,v_s) {:}{=}\left( \frac{\alpha }{5}, 1 + \frac{\alpha ^2}{25}\right)$, where the Jacobian is given by

$$\begin{pmatrix} f_u & f_v \\ g_u & g_v \end{pmatrix} {:}{=}\frac{1}{\alpha ^2 + 25} \begin{pmatrix} 3\alpha ^2 - 125 & -20\alpha \\ 2\alpha ^2 \beta \sigma & -5\alpha \beta \sigma \end{pmatrix},$$

we obtain the conditions

C1 $$\begin{aligned} f_u + g_v < 0, \end{aligned}$$

C2 $$\begin{aligned} f_u g_v - f_v g_u > 0, \end{aligned}$$

C3 $$\begin{aligned} \delta \sigma f_u + g_v > 0, \end{aligned}$$

C4 $$\begin{aligned} \left( \delta \sigma f_u + g_v \right) ^2 - 4 \delta \sigma \left( f_u g_v - f_v g_u \right) > 0. \end{aligned}$$

Combining all four inequalities (C1-C4) defines a region of parameter space, called the *Turing space*, where the system can give rise to spatially heterogeneous stable patterns. As motivation for the following section, the two following subsections explore some of the classical linear and weakly nonlinear techniques used to analyse Turing systems, and their limitations.

### Linear Analysis of the CIMA System

We start by recalling that (C4) is obtained by considering the conditions under which *h*, defined by

$$\begin{aligned} h(k^2) {:}{=}\delta \sigma k^4 - (\delta \sigma f_u + g_v)k^2 + (f_u g_v - f_v g_u), \end{aligned}$$

has real roots $k^2_- \le k^2_+$, introducing a (possibly empty) set of integers $k^2$ where $h\left( k^2\right) < 0$, leading to unstable eigenfunctions that grow exponentially in $\tau$. The linear theory therefore predicts that the behaviour of the solution is eventually dominated by the summand associated to the maximal wavenumber, $k_{\max } = \mathop {\mathrm {arg\,max}}\limits {\lambda (k^2)}$. Here we have implicitly used a standard assumption, which is sensible for any physically realisable system, that the initial condition will excite all modes, as this is only avoided with precision fine-tuning. To account for this, we remark that later in the paper, when solving (CIMA) numerically with a finite-element method, we choose initial conditions where the value at each vertex in the mesh is an independent identically distributed Gaussian $\textrm{N}\left( 0,0.1^2\right)$ perturbation of the steady state $(u_s, v_s)$.

Next, we note that for $\xi = (x,y)$ in the domain $\Omega = [0,L_x] \times [0,L_y]$ with Neumann boundary conditions, the eigenvalue-eigenfunction solutions $(k, \boldsymbol{S}_k)$ are given by

3 $$\begin{aligned} \begin{aligned} k_{m,n}^2&= \frac{\pi ^2 m^2}{L_x^2} + \frac{\pi ^2 n^2}{L_y^2},\\ \boldsymbol{S}_{k_{m,n}}&= c_{m,n} \operatorname {cos}\left( \frac{m \pi x}{L_x}\right) \operatorname {cos}\left( \frac{n \pi y}{L_y}\right) . \end{aligned} \end{aligned}$$

Here (*m*, *n*) are integer pairs, not both zero, and $c_{m,n}$ are constants. For each such pair, we can determine the subregion of the Turing space within which the mode $\boldsymbol{S}_{k_{m,n}}$ is stable by considering the sign of $\lambda (k_{m,n}^2)$ for those $k_{m,n}$ where $h(k_{m,n}^2) < 0$. We then are able to quantify the number of modes that are unstable at a point in parameter space as $\left| \left\{ (m,n) \in \mathbb {Z}^2 :h(k_{m,n}^2)< 0 < \lambda (k_{m,n}^2)\right\} \right|$.

**Fig. 1:**
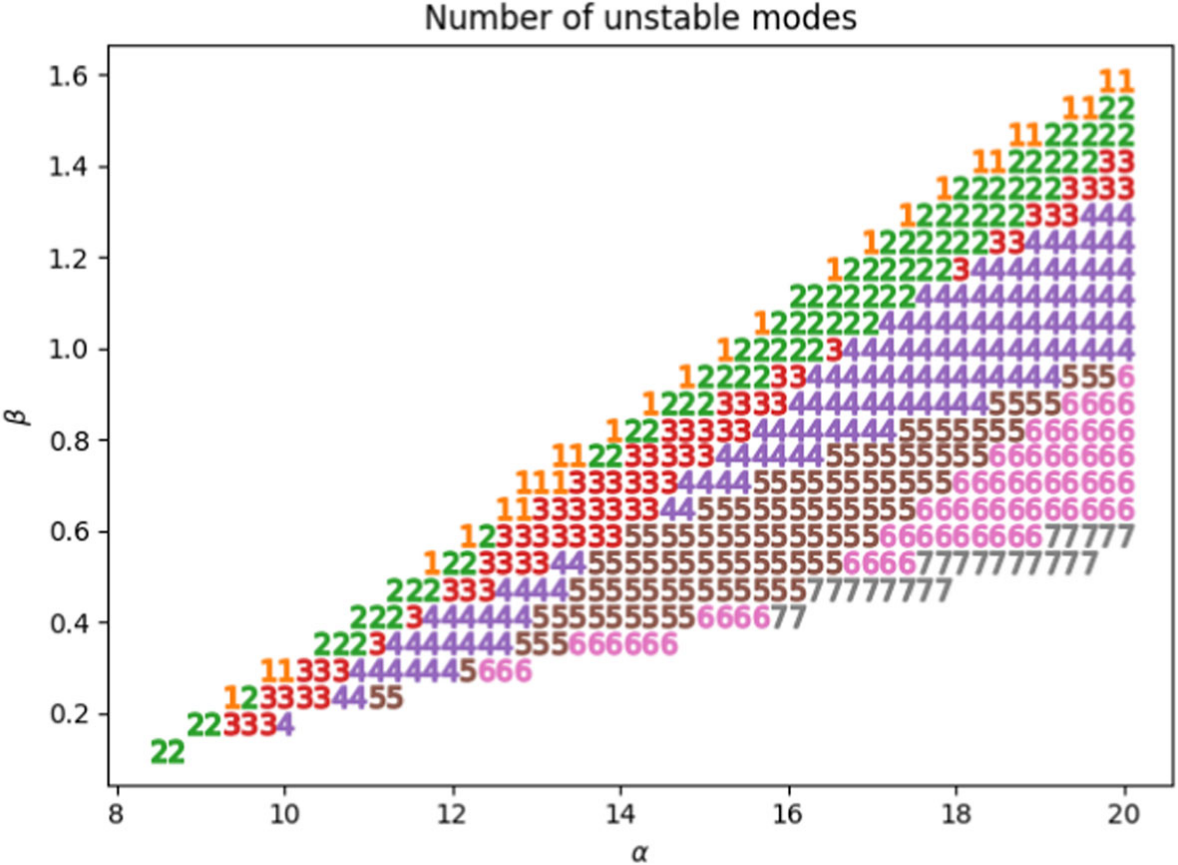
Number of unstable Fourier modes for $n=0$ and $m \in \{0,1,...,10\}$, with $\alpha$-$\beta$ axes and fixed parameters $\sigma = 20, \delta = 1.5, L_x = L_y = 20$

As shown in Figure 1, even for a fixed *n*, there are multiple unstable modes for a range of parameters values. We note that once the nonlinear terms begin to play a significant role in the dynamics, we expect the solutions to depend (albeit weakly) on all the excited unstable modes. Outside a relatively small subset of the Turing space where there is a unique unstable mode, there is sensitivity to certain initial conditions (such as ours, where the initial condition is a white noise perturbation of the steady state that excites all modes) due to mode selection, a well-known difficulty with Turing systems (Arcuri and Murray 1986). Furthermore, we remark that unlike at the upper boundary of the Turing space in Figure 1 (where $\left( \delta \sigma f_u + g_v \right) ^2 - 4 \delta \sigma \left( f_u g_v - f_v g_u \right) = 0$), at the lower boundary (which corresponds to the Hopf bifurcation that occurs at $f_u + g_v = 0$), the number of unstable modes remains bounded away from 1 as we observe unstable solutions at and beyond the bifurcation.

Returning to an idea of Arcuri and Murray (1986), we consider the mode with the largest eigenvalue $\lambda (k_{m^*,n^*}^2)$ and examine the parameter dependence of $\left( m^*,n^*\right)$ in the following figures.

**Fig. 2:**
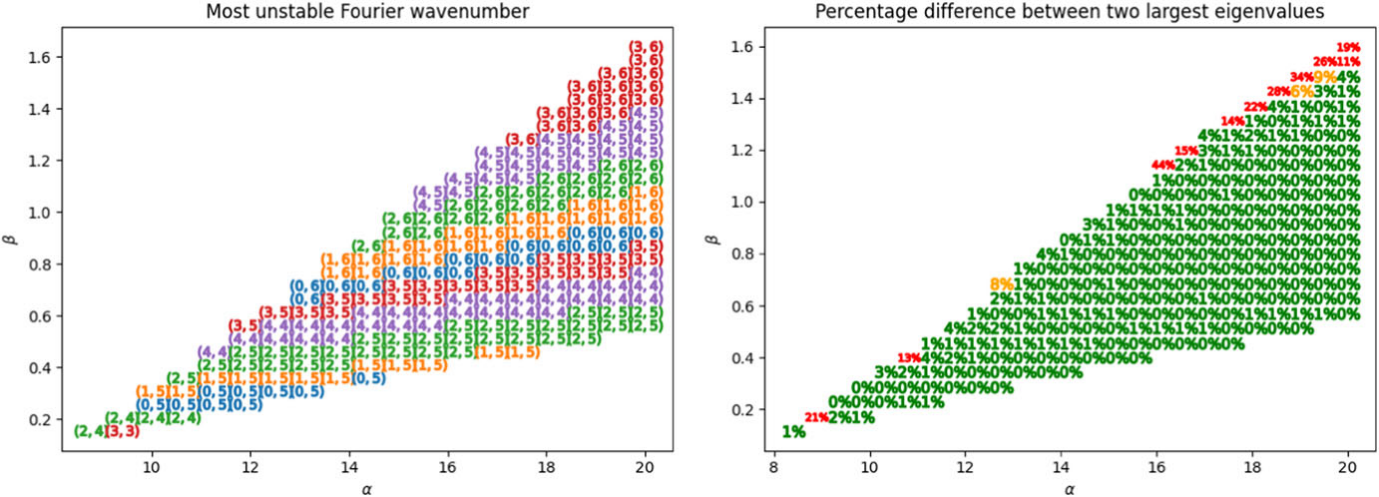
Wavenumbers $(m^*,n^*)$ associated to the most dominant mode (left), and percentage difference between the two largest eigenvalues with points coloured according thresholds of $5\%$ and $10\%$ (right). Parameters are fixed at $\sigma = 20, \delta = 1.5, L_x = L_y = 20$

As shown to the left in Figure 2, we observe significant heterogeneity in the linearly dominant modes across the Turing space, but remark that the differences in magnitude between the first largest and the second largest eigenvalues are small, as shown to the right in Figure 2. Since the solutions to (CIMA) are bounded, this suggest that multiple modes contribute to the final pattern arising from the nonlinear dynamics, and that the initial condition also plays a significant role in this patterning.

In light of this classically-inspired analysis, we conclude, as others have demonstrated in general, that the linear theory is not sufficient to achieve our primary objective of quantifying the parameter dependence of the emergent patterns in the CIMA system. We turn to another classical analysis that circumvents the need for more sophisticated tools, like topological data analysis, to classify patterns, albeit with strong restrictions on the domain’s geometry.

### Weakly Nonlinear Analysis of Turing Systems: Pattern Selection on Small Domains

In Ermentrout (1991), a mechanism for selection of spots or stripes in general reaction diffusion systems is described. The mechanism demonstrates that the emergence of spots or stripes is *not* determined by linear effects – Ermentrout does this by explicitly constructing an activator-inhibitor system where a quadratic perturbation changes the pattern from stripes to spots, demonstrating that analyses of the linear spectrum are not sufficient to determine the emergent patterns. Furthermore, Ermentrout proves a necessary and sufficient criterion for the selection of stripes or spots in terms of the quadratic and cubic terms of the reaction diffusion system when the domain is a sufficiently small square with *periodic* boundary conditions.

In particular, the results are specific to the regime where the domain $\Omega$ is small enough so that the first unstable mode has wavenumber $k \in \{k_{0,1}, k_{1,0}, k_{1,1}\}$, and all other modes are stable. The criterion defines two quantities *a* and *b*, such that the stripe solution ($k \in \{k_{0,1}, k_{1,0}\}$) is stable iff $b< a < 0$, and the spot solution ($k = k_{1,1}$) is stable iff $a< -|b| < 0$. In our case, these evaluate to

$$\begin{aligned}&a= \frac{1875 \delta \sigma }{\left( \alpha ^2-75\right) ^2 \left( \alpha ^2 (2 \delta \sigma -3)+225\right) } \times \frac{28 \alpha ^4-5175 \alpha ^2-39375}{7 \alpha ^2+675}, \\ &b= \frac{1875 \delta \sigma }{\left( \alpha ^2-75\right) ^2 \left( \alpha ^2 (2 \delta \sigma -3)+225\right) } \times \frac{3 \left( 11 \alpha ^6-875 \alpha ^4-119375 \alpha ^2+234375\right) }{\left( \alpha ^2+25\right) \left( \alpha ^2+525\right) }. \end{aligned}$$

We observe that the only terms containing $\delta$ or $\sigma$ are common to both *a* and *b* so, along the bifurcation, we obtain critical points $\alpha _1$, $\alpha _2$, and $\alpha _3$ (equal to 2.501, 11.581, 12.630 to 3 d.p. respectively) such that the stripe solution is stable iff $\alpha \in (\alpha _1,\alpha _2)$ and the spot solution is stable iff $\alpha \in [0,\alpha _1) \cup (\alpha _2,\alpha _3)$. Although this progresses our knowledge of the parameter dependence of emergent patterns in the CIMA system, the analysis remains limited to very specific cases in the vicinity of the simplest bifurcation, and does not necessarily carry over to other domains, such as larger domains, or ones with non-periodic boundary conditions.

Having given this very brief review of some classical linear and weakly nonlinear techniques, we see that classifying the patterns that emerge from the CIMA system could strongly benefit from techniques that are applicable in the fully nonlinear regime. To progress further, we turn to rigorously introducing the machinery of algebraic topology that will be employed in later sections.

## Background on Algebraic Topology for Data Analysis

This section introduces necessary background about the main tool in topological data analysis – persistent homology – which underlies the methods we will use to study the CIMA system. For an overview geared towards applications, see the roadmap by Otter et al. (2017), and also lecture notes by Nanda (2024). For the full technical details, we require a basic familiarity with constructions of classical algebraic topology; the reader is directed to (Hatcher (2002), Chapter 2) for a thorough introduction to the topic. We quantify the spatial patterns arising from the CIMA and Schnakenberg kinetics using the main tool in topological data analysis, persistent homology. There are many introductions to topological data analysis (Edelsbrunner and Harer 2010; Ghrist 2014; Carlsson 2009), with foundations of algebraic topology (Hatcher 2002, Chapter 2), computational algebraic topology lecture notes (Nanda 2024) as well as material assuming only linear algebra (Otter et al. 2017; Schenck 2022). The data setting here takes in a mesh with vertices, given by the solution of the PDE at that point, encoded as real-valued functions *u* and *v* representing the chemical concentration of reactants *u* and *v*. The idea behind persistent homology, which we apply here, is to study how the topology (homology in degree 0 and 1, i.e., connected components and loops) of these data change along a height function. These topological computations are across the values of *u* and *v*, use algebra to measure the persistence of topological features. The multiscale data analysis pipeline outputs a barcode for reactants in each dimension, which we describe in the next subsection. The attraction of persistent homology for data applications originates from the celebrated stability theorems, which informally state that small perturbations in the input data result in small perturbations of the given barcode, resulting in a robust tool for analysing noisy data. As we describe in the following subsection, one can also equip the space of barcodes with a metric, allowing comparisons between the topological summaries of two points in the mesh.

### Data, Filtrations and Barcodes

To motivate some of the following definitions, we draw attention to the dataset we will apply the techniques of the following section to. The system of PDEs in question (in our case, the CIMA or Schnakenberg system) is solved numerically on a mesh, giving real-valued functions *u* and *v*, whose values are only computed at the vertices of the mesh. In our work, the PDEs will be obtained from the CIMA or Schnakenberg system, and the mesh will be a triangulation of the rectangle $\Omega = [0,L_x] \times [0,L_y]$. We begin by defining a few building blocks.

Let *V* be a finite set; in our setting *V* will be the set of mesh vertices.

#### Definition 3.1

A *finite simplicial complex* *K* is a collection of subsets of *V* called *simplices*, such that for every $v \in V$, we have $\{v\} \in K$, and whenever $\sigma \in K$ and $\tau \subset \sigma$, we also have $\tau \in K$. The *dimension* $\textrm{dim} (\sigma )$ of a simplex $\sigma$ is given by $|\sigma | - 1$, and a *d*-dimensional simplex is often referred to as a *d*-simplex.

The first step towards quantifying the shape of the solutions *u* and *v* is to view appropriate subsets of the simplicial complex *K* that we obtain by triangulating $\Omega$. In particular, we define a *subcomplex* as follows.

#### Definition 3.2

Let *K* be a finite simplicial complex. We say a subset $L \subset K$ is a *subcomplex* of *K* if the following holds: for each $\tau \in L$, if $\sigma$ is a simplex of *K* whose vertices are all vertices of $\tau$, then $\sigma \in L$.

#### Example 3.3

The *standard 2-simplex* is the simplicial complex, $\Delta (2)$, whose vertex set *V* is given by $V = \{0,1,2\}$. The simplices of $\Delta (2)$ are $\{0\},\{1\},\{2\},\{0,1\},\{1,2\},\{2,0\},\{0,1,2\}$. The *standard 1-simplex*, $\Delta (1)$ whose simplices are $\{0\},\{1\},\{0,1\}$, can be seen as a subcomplex of $\Delta (2)$. $\Delta (1)$ and $\Delta (2)$ are visualised as the first and fourth simplicial complexes in Example 3.7, respectively.

Nesting subcomplexes of a simplicial complex will prove instrumental in extracting useful topological features of our data, which we do using *filtrations*.

#### Definition 3.4

Let *K* be a finite simplicial complex. A *filtration* $\mathcal {F}_\bullet K$ of *K* is a sequence of subcomplexes $\mathcal {F}_j K$ of *K* and inclusions $\iota _{i \rightarrow j} :\mathcal {F}_i K \hookrightarrow \mathcal {F}_{j} K$ between any pair of filtration values $i<j$. We also require the existence of some filtration value $j^*$ such that $\mathcal {F}_{j^*} K = K$, so that the filtration is *exhaustive*.

We will be interested in filtrations induced by the sublevel sets of the continuous functions *u* and *v*.

#### Definition 3.5

Let *X* be a nonempty topological space and $f :X \rightarrow \mathbb {R}$ be continuous. The *sublevel set filtration* of *X* induced by *f* is the filtration given by $f_{\le t} X {:}{=}f^{-1}(-\infty , t]$, together with the natural inclusions $\iota _{s \le t} :f_{\le s}X \hookrightarrow f_{\le t}X$ for $s \le t$.

#### Remark 3.6

We note that replacing *f* by its negative $-f$ gives an equivalent *superlevel set filtration*, and that the theoretical results that follow in Subsection 3.2 hold completely analogously for superlevel set filtrations.

Once we have our filtration, we describe the connectivity of *u* and *v* across the filtration using a computable topological invariant, homology. Given a simplicial complex at a filtration value of *u* (or *v*), we compute the rank of homology groups (which are quotient vector spaces whose dimension gives the number of connected components, loops, and higher-dimensional voids of a space). The induced maps between them will be key to quantifying the topology of the solutions obtained from the PDEs. In particular, at each degree $k \ge 0$, taking homology with coefficients in a field $\mathbb {F}$ guarantees that the homology groups will be endowed with the structure of a vector space and yields the sequence $\operatorname {H}_k \left( \mathcal {F}_\bullet K \right)$. Functoriality of homology naturally allows us to define linear maps between $\operatorname {H}_k \left( \mathcal {F}_i K \right)$ and $\operatorname {H}_k \left( \mathcal {F}_j K \right)$ given by $\operatorname {H}_k \iota _{i \rightarrow j}$.

Computing homology of a single filtration (e.g. given by level sets of *u* or *v*) is encoded in a one-parameter persistence module. This algebraic object can be decomposed, for the finite data considered here, in a manner given by a structure theorem (Zomorodian and Carlsson 2005). The output of these algebraic topology computations is a multiset of intervals, called the *barcode*, and denoted by $\operatorname {Bar}\left( \operatorname {H}_k (\mathcal {F}_\bullet K), \operatorname {H}_k \iota _\bullet \right)$ for each dimension $k \ge 0$. Each interval or bar representing a topological feature (from the homology computations) present in the data, such as a connected component or a loop, that appears at a filtration value called the *birth* value, $b(\gamma )$, of the feature and disappears (or *dies*) at a filtration value, referred to as the *death* value, $d(\gamma )$. When the feature, such as a connected component never disappears, the convention is to set $d(\gamma ) = \infty$. A barcode or persistence diagram is a set of birth/death pairs that is either visualised as a multiset of intervals or plotted as (*x*, *y*) coordinates $\left( b(\gamma ),d(\gamma )\right)$ where all points are above the diagonal. Long bars or points that are far from the diagonal are called *persistent*.

For discrete filtrations, algorithms at our disposal allow us to compute the persistent homology groups at every degree *k* simultaneously (see Edelsbrunner and Harer (2010), Chapter VII.2, for example). Our implementation uses the persistence() method implemented in The GUDHI Project (2021), originally due to Dey et al. (2014); Boissonnat et al. (2013); de Silva et al. (2011b). The structure theorem, together with the availability of packages implementing the algorithm, practically allows us to uniquely represent the persistent homology groups as their barcodes.

#### Example 3.7

Consider the simplicial complex $K = \Delta (2)$, and the discrete filtration $\mathcal {F}_\bullet K$ given below.
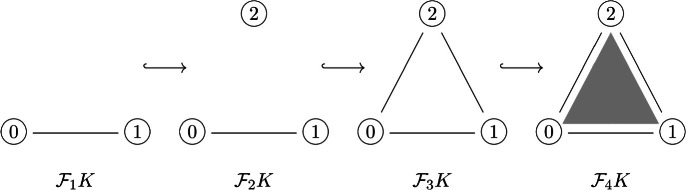


Writing $B_k$ for the barcodes $\operatorname {Bar}\left( \operatorname {H}_k (\mathcal {F}_\bullet K), \operatorname {H}_k \iota _\bullet \right)$ for each $k \ge 0$, and carrying out the standard algorithm for computing barcodes yields $B_0 = \left\{ [1,\infty ), [2,3] \right\}$ and $B_1 = \left\{ [3,4]\right\}$.

We can see this indeed agrees with the persistent homology groups as follows. In degree 0, the generator of homology $\gamma _0$ associated to the connected component of the simplex {0} continues to be a nontrivial generator under each of the inclusions $\operatorname {H}_0 \iota _{1 \rightarrow j}$, so has $(b(\gamma _0),d(\gamma _0)) = (1,\infty )$, giving the barcode $[1,\infty )$. On the other hand, the generator $\gamma _2$ of homology associated to {2} has $\operatorname {H}_0 \iota _{2 \rightarrow 3}(\gamma _2) = \operatorname {H}_0 \iota _{1 \rightarrow 3}(\gamma _0)$, as the two generators differ by the simplicial boundary of the 1-simplex $\{0,2\}$. Since $\gamma _0$ appeared as a generator in an earlier filtration value, $\gamma _2$ does not contribute to homology at filtration value $j=3$, giving the barcode [2, 3].

In degree 1, the first nontrivial generator appears at filtration value $j=3$ in the form of the loop $\gamma _{012} = \{0,1\} + \{1,2\} - \{0,2\}$, but this is trivialised under $\operatorname {H}_1 \iota _3$ as it is the boundary of the 2-simplex $\{0,1,2\}$, yielding the barcode $[b(\gamma _{012}), d(\gamma _{012})] = [3,4]$.

We also present a visualisation of barcodes as follows.

#### Definition 3.8

(Cohen-Steiner et al. (2007)) A *persistence diagram* is a multiset $D =\operatorname {Dgm}\left( \operatorname {H}_k (\mathcal {F}_\bullet K), \operatorname {H}_k \iota _\bullet \right)$ in $\mathbb {R} \times \left( \mathbb {R} \cup \{\infty \}\right)$ whose points are the elements of $\operatorname {Bar}\left( \operatorname {H}_k (\mathcal {F}_\bullet K), \operatorname {H}_k \iota _\bullet \right)$ (counted with multiplicity), together with the diagonal $\Delta = \left\{ (b,b) \in \mathbb {R} \times \left( \mathbb {R} \cup \{\infty \}\right) \right\}$, counted with infinite multiplicity.

#### Remark 3.9

It is immediate from the definition that persistence diagrams contain the same information as barcodes, only embedded into $\mathbb {R} \times \left( \mathbb {R} \cup \{\infty \}\right)$. Since barcodes capture the birth and death filtration values of homology generators by definition, persistence diagram axes are typically labelled accordingly.

#### Example 3.10

**Fig. 3:**
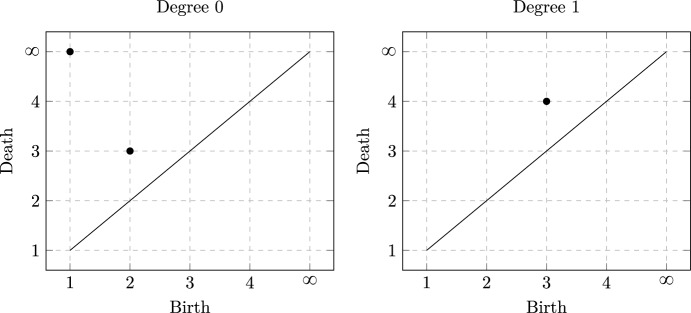
Persistence diagrams of the discrete filtration from Example 3.7 in degrees 0 and 1. Left: the point $(1,\infty )$ represents the connected component associated to $\{0,1\}$, which persists for all filtration values; while the point (2, 3) represents the connected component associated to $\{2\}$, which appears at filtration value $j=2$ and merges with that of $\{0,1\}$ at filtration value $j=3$. Right: The point (3, 4) represents the loop $\gamma _{012}$ that appears at filtration value $j=3$, which persists until filtration value $j=4$, where it becomes contractible

We can reformulate the barcodes $B_0 = \left\{ [1,\infty ), [2,3] \right\}$ and $B_1 = \left\{ [3,4]\right\}$ from Example 3.7 as the persistence diagrams in Figure 3.

### The Stability Theorems

To justify the use of persistent homology as the basis for topological data analysis, one would like a robust shape descriptor of the data. A result known as the *stability theorem* theoretically guarantees the robustness of barcodes to changes in the input data. To quantify this precisely, we require a few more definitions, including the alternative visualisation of barcodes as persistence diagrams. To state the stability theorem, we endow the space of persistence diagrams with a family of metrics that are pointwise induced by the embedding into $\mathbb {R} \times \left( \mathbb {R} \cup \{\infty \}\right)$.

#### Definition 3.11

Let $D_1, D_2$ be two persistence diagrams. We say a map $\mu$ is a *matching* between $D_1$ and $D_2$ if it is a bijection $D_1\cup \Delta \rightarrow D_2\cup \Delta$. It is standard to abuse notation and write $\mu :D_1 \rightarrow D_2$ to notate such a matching, with the understanding that points of each persistence diagram can be paired with points along the diagonal $\Delta$.

Let $1 \le p,q \le \infty$, then define the *q-Wasserstein distance* between two diagrams as

$$\begin{aligned} W_{p,q}(D_1,D_2) {:}{=}\inf _{\mu :D_1 \rightarrow D_2} \left( \sum _{x \in D_1} \Vert x - \mu (x) \Vert _p^q\right) ^{\frac{1}{q}}, \end{aligned}$$

interpreting the $q = \infty$ case as

$$\begin{aligned} W_{p,\infty }(D_1,D_2) {:}{=}\inf _{\mu :D_1 \rightarrow D_2} \sup _{x \in D_1} \Vert x - \mu (x) \Vert _p, \end{aligned}$$

and adopting the convention $\infty - \infty = 0$ to ensure it is well-defined for infinite death times.

#### Remark 3.12

We briefly note that the space of persistence diagrams with the *q*-Wasserstein distance is a complete separable metric space, allowing a rigorous treatment of means, variances, and other probabilistic quantities (Mileyko et al. 2011).

With this metric in hand, we have half of the machinery necessary to state a version of the stability theorem. The other half is related to the input data via the choice of filtration of the simplicial complex. We preface the following with the fact that all our input data can be treated as coming from tame functions, therefore enjoying the privileges of Theorem 3.15.

#### Definition 3.13

Let *X* be a topological space and $f :X \rightarrow \mathbb {R}$ be continuous. We say *f* is *tame* if for all $k\ge 0$, the vector spaces $\operatorname {H}_k \left( f_{\le t}X \right)$ are finite-dimensional for all *t*, and there exist only finitely many $t_i$ that admit no $\varepsilon > 0$ for which $\operatorname {H}_k \iota _{t_i - \varepsilon \le t_i + \varepsilon }$ is an isomorphism. Such $t_i$ are called *homological critical values* of *f*.

#### Remark 3.14

Tame functions *f* allow us to reduce the $\mathbb {R}$-indexed persistence module $\left( \operatorname {H}_k \left( f_{\le t}X\right) , \operatorname {H}_k \iota _{s \le t}\right)$ to a persistence module of finite type. We do so by extracting the sequence $t_1< t_2< \dots < t_n$ of homological critical values of *f*, and pick filtration values $-\infty = j_0< t_1< j_1< t_2< \dots< j_{n-1}< t_n < j_n = \infty$ so that $\operatorname {H}_k \left( f_{\le j_\bullet }X\right)$ now recovers the persistent homology of *X* with respect to $f_{\le t}X$.

Denoting $\operatorname {Dgm}\left( \operatorname {H}_k(f_{\le t}K), \operatorname {H}_k \iota _{s \le t}\right)$ by $\operatorname {Dgm}_k(f)$, we can now state a version of the stability theorem due to Cohen-Steiner, Edelsbrunner and Harer as follows.

#### Theorem 3.15

(Cohen-Steiner et al. (2007)) Let *K* be a finite simplicial complex, let $f,g :|K| \rightarrow \mathbb {R}$ be tame functions, and let $k \ge 0$, then

$$\begin{aligned} W_{\infty ,\infty }(\operatorname {Dgm}_k(f),\operatorname {Dgm}_k(g)) \le \Vert f - g \Vert _\infty . \end{aligned}$$

Here |*K*| denotes the geometric realisation of *K*. In fact, under the slightly stronger conditions that *f* and *g* are Lipschitz continuous, and that the minimum number of simplices *N*(*r*) in a triangulation of *K* with mesh *r* *grows polynomially* (i.e. that there exist constants *c* and *j* such that $N(r) \le \frac{c}{r^j}$), Cohen-Steiner et al. went on to prove another version of the stability theorem for the *q*-Wasserstein distances (with $p = \infty$) between $\operatorname {Dgm}_k(f)$ and $\operatorname {Dgm}_k(g)$ for all sufficiently large *q* Cohen-Steiner et al. (2010); Edelsbrunner and Harer (2010).

The stability theorems demonstrate that perturbing the input function by a small amount also perturbs the persistence diagram by a small amount. In particular, points in $\operatorname {Dgm}_k(f)$ sufficiently far from the diagonal (or equivalently, points corresponding to long barcodes) are stable. We conclude by noting that the classical stability theorems of Theorem 3.15 have analogues for filtrations obtained from geometric filtered complexes which can be used in more general applications.

## Pipeline of Topological Clustering of Turing Patterns

In this section, we outline the tools used to analyse data obtained from numerical simulations of the CIMA system, with the aim of clustering points in the Turing space depending on the final stable pattern; we then briefly outline a topological framework for parameter estimation in Turing systems.

### Numerical Simulation of PDEs

To implement the persistent (co)homology algorithm, direct numerical simulation of the nonlinear reaction diffusion systems is required. We opt to use Python’s py-pde library primarily due to three features, namely adaptive timestepping and (numerical) steady state detection for the explicit solver, as well as the availability of an implicit solver. Zwicker (2020).

Given the numerical solution of a PDE on a mesh, the triangulation on this mesh allows us to calculate the simplicial homology of various subsets of the solution. However, it is not immediately obvious why the aforementioned homological construction is necessary to study Turing patterns— we shed some light on this in the remainder of this section.

#### Remark 4.1

To proceed, we must start with a sufficiently fine triangulation of the domain $\Omega$ that captures the large spatial gradients associated with patterns, while ensuring it does not contain too many vertices so as to make the PDE solver’s runtime unreasonable. One ad hoc method of obtaining an upper bound, inspired by the classical Nyquist–Shannon sampling theorem, is selecting a triangulation whose simplices have a diameter no larger than half the minimal unstable wavelength.

We progress by selecting a subset of parameter space where the diversity of the final stable patterns exhibited by the CIMA system (CIMA) is observed.

### Domain, Parameter Space and Filtration Discretisation

Begin by fixing the domain $\Omega$ to be the square of sidelength $L_x = L_y = 20$. We require intervals of $\sigma$ (which scales with the starch concentration) and $\alpha$ (the nondimensionalised production of iodide), noting that bounding $\sigma$ and $\alpha$ results in a bounded subset of the Turing space with no other restrictions necessary on $\beta$; the bounds for $\sigma$ were chosen as $\sigma \in [1, 20]$. Given this bound on $\sigma$, numerical experiments confirmed that the patterns observed by bounding $\alpha$ above by 20 showed sufficient diversity between stripes, spots and labyrinths, so the bounds for $\alpha$ were chosen as $\alpha \in [0, 20]$. Qualitatively, the domain size strikes a balance between being sufficiently large, so that multiple modes are unstable, and requiring a feasible amount of computation time to solve (CIMA) numerically when meshed to a fineness dictated by Remark 4.1.

Following Remark 4.1, we seek the unstable mode with the smallest wavelength within this subset of the Turing space, which turns out to be the mode associated to the wavenumber (9, 0). We therefore require the maximal spatial discretisation stepsize to be at most $\texttt {stepsize} = 1.0$, which is slightly smaller than half the minimal wavelength. It was computationally feasible to choose a finer discretisation, so we fixed $\texttt {stepsize} = 0.5$. Next, we triangulate this restricted Turing space with 549 vertices (which we will henceforth refer to as *nodes*) to capture the parameter dependence of the data, but also small enough so the computations terminate in a reasonable amount of time. Numerical experiments confirmed that neither decreasing $\texttt {stepsize}$ nor locally increasing the number of nodes affected the conclusions.

We are forced, given finite computational power, to fix $\texttt {stepsize}$ and the maximal solver runtime, meaning our notion of convergence is restricted to *computational* convergence. To allay this, we confirm the clustering in Subsection 4.3 does not substantially change when doubling the runtime, or reducing $\texttt {stepsize}$ so the number of gridpoints is doubled. While this does not rule out the fact that some of the observed patterns may be transient, it does allow us to conclude that the resolution is sufficient to capture the parameter-dependence we are interested in. We remark that for some nodes, such as those near the boundary of the Turing space, the real part of the eigenvalues (of the stable modes) can be arbitrarily small in magnitude, meaning a numerical solver will have arbitrarily long runtime before converging. However, we can set the numerical resolution to one where we can capture the parameter-dependence we seek, noting that in biochemical systems like the CIMA system, it is primarily these observable intermediate-time patterns that are of interest, given physical timescales. In our simulations, we evolve the PDEs until $\tau = 250$.

Finally, the CIMA system (CIMA) is solved at each node until convergence to a stable pattern, giving a finite simplicial complex *K* endowed with functions $u :|K| \rightarrow \mathbb {R}$ and $v :|K| \rightarrow \mathbb {R}$ whose values are known at vertices of the triangulation of *K*. Two filtrations, a lower-star and upper-star filtration $\mathcal {F}_1 K \hookrightarrow \dots \hookrightarrow \mathcal {F}_{20} K$ approximating the sublevel sets $u_{\le \min u + \frac{1}{20}\left( \max u - \min u\right) j} |K|$ and superlevel sets $v_{\ge \max v - \frac{1}{20}\left( \max v - \min v\right) j} |K|$ of *u* and *v* respectively, are taken as

4 $$\begin{aligned} \begin{aligned} \mathcal {U}_j K&= \left\{ \sigma \in K :u(|\tau |) \le {\min u + \frac{\max u - \min u}{20}j} \; \text {for each 0-simplex } \tau \; \text {of } \sigma \right\} , \\ \mathcal {V}_j K&= \left\{ \sigma \in K :v(|\tau |) \ge {\max v - \frac{\max v - \min v}{20}j} \; \text {for each 0-simplex } \tau \; \text {of } \sigma \right\} . \end{aligned}\end{aligned}$$

Barcodes for the persistent cohomology groups of *K* with respect to these filtrations are calculated using the Python implementation of GUDHI’s persistence (The GUDHI Project 2021). Importantly, we observe that the fact we are working with fields (in our case $\mathbb {F}_p$ for *p* prime) means that the universal coefficients theorem guarantees the equivalence of the barcodes obtained from persistent cohomology and the dual persistent homology (Hatcher 2002; de Silva et al. 2011a). Finally, we note that this choice of filtration also naturally normalises the data, allowing for a direct comparison of the barcodes.

### Topological Clustering Algorithm

Aiming to classify the final stable patterns of solutions to the CIMA system into “stripes", “spots" or “labyrinths", we implement a hierarchical clustering algorithm.

At each node $\theta _i$ in the discretised Turing space, we now have four multisets of intervals – $B_j^u(\theta )$ and $B_k^v(\theta )$ corresponding to the barcodes for *u* in dimensions $j = 0,1$, and for *v* in dimensions $j = 0,1$ respectively. Using the 2-Wasserstein distance $W_{2,2}$ (see Definition 3.11), we can endow the Turing space with a metric *d* given by the sum of $W_{2,2}$ distances between respective barcodes, i.e.

5 $$\begin{aligned} d\left( \theta _1, \theta _2\right) {:}{=}\sum _{\begin{array}{c} j \in \{0,1\} \\ w \in \{u,v\} \end{array}} W_{2,2}\left( B_j^w(\theta _1) ,B_j^w(\theta _2)\right) . \end{aligned}$$

Equipped with this metric, we can now carry out hierarchical clustering with various choices for the maximum number of clusters. We also compared the performance of different cluster linkage methods on the data by computing the silhouette score (Rousseeuw 1987) of the various clusterings that are produced. For each PDE, the chosen linkage method and number of clusters were the ones that gave the highest silhouette score.

The clustering was performed using SciPy (Virtanen et al. 2020) and silhouette scores were computed using scikit-learn (Pedregosa et al. 2011). To verify that the clustering produces clusters that capture the features of interest (“stripes", “spots", “labyrinths"), we sample images from a selection of nodes to ensure inter-cluster agreement and intra-cluster diversity. This is also globally quantified using the aforementioned silhouette scores.

We turn to an application of the clustering algorithms to the CIMA and Schnakenberg systems in the following section.

## Results

This section begins by showing our results for the CIMA system, before turning to a comparison with the Schnakenberg system.

To start, we carry out the methodology in Subsection 4.2 to obtain barcodes at the nodes of the discretised (restricted) Turing space and offer a simple observation about the distribution of the length of barcodes, with a chemically or biologically relevant interpretation briefly discussed in Subsection 5.2. Similarly to Subsection 4.3, we employ the notation $B_j^u {:}{=}\operatorname {Bar}\left( \operatorname {H}_j (\mathcal {U}_\bullet K), \operatorname {H}_j \iota _\bullet \right)$ and $B_j^v {:}{=}\operatorname {Bar}\left( \operatorname {H}_j (\mathcal {V}_\bullet K), \operatorname {H}_j \iota _\bullet \right)$.

**Fig. 4:**
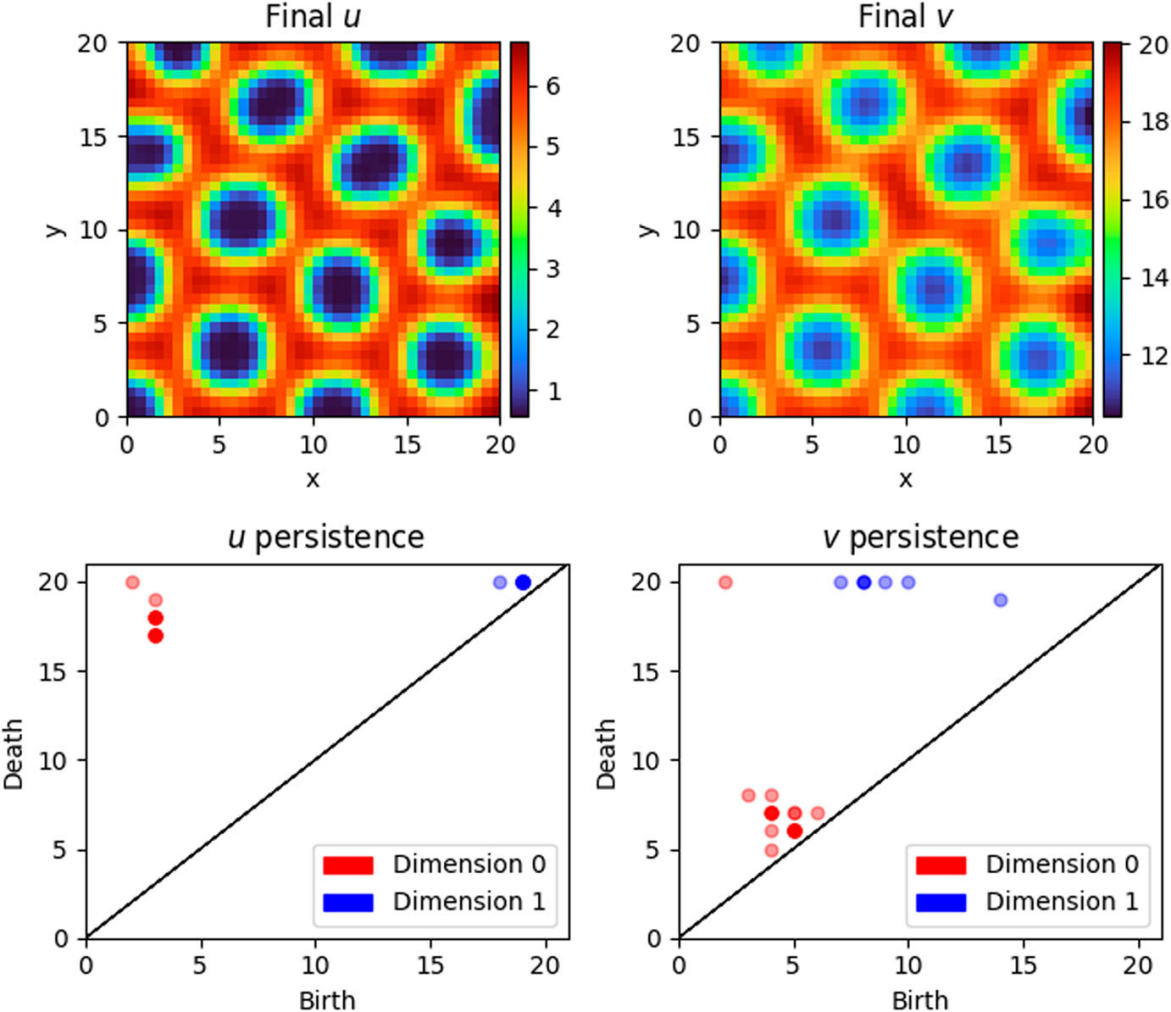
A realisation of the CIMA system (CIMA) for the conditions described in Subsection 4.2, with persistence diagrams for *u* and *v*. Here $\alpha = 9.74$, $\beta = 0.27$, $\delta = 1.5$ and $\sigma = 12.5$

Figure 4 shows typical realisations and persistence diagrams for a spots pattern. The bottom-left persistence diagram has multiple generators of homology in degree 0 (connected components) that persist for many lower-star filtration values, represented by red points. Each generator corresponds to a minimum in the final concentration of *u*, capturing a single spot. The spots in the interior of $\Omega$ are also captured by the persistent homology of the final concentration of *v*: the bottom-right persistence diagram has multiple generators of homology *in degree 1* (loops) that persist for many *upper-star* filtration values, represented by *blue* points. Intuitively, each generator here corresponds to a loop around a minimum in the final concentration of *v*.

As shown in Figure 4, calculating the barcodes for a typical spots pattern, we observe that the homology of the filtration is invariant for a long sequence of filtration values. For example for *u*, in degree 0, the homology groups $\operatorname {H}_0 \left( \mathcal {U}_j K \right)$ are isomorphic between filtration values $j=3$ and $j=17$; and in degree 1, the groups $\operatorname {H}_1 \left( \mathcal {U}_j K \right)$ are isomorphic between filtration values $j=0$ and $j=18$.

**Fig. 5:**
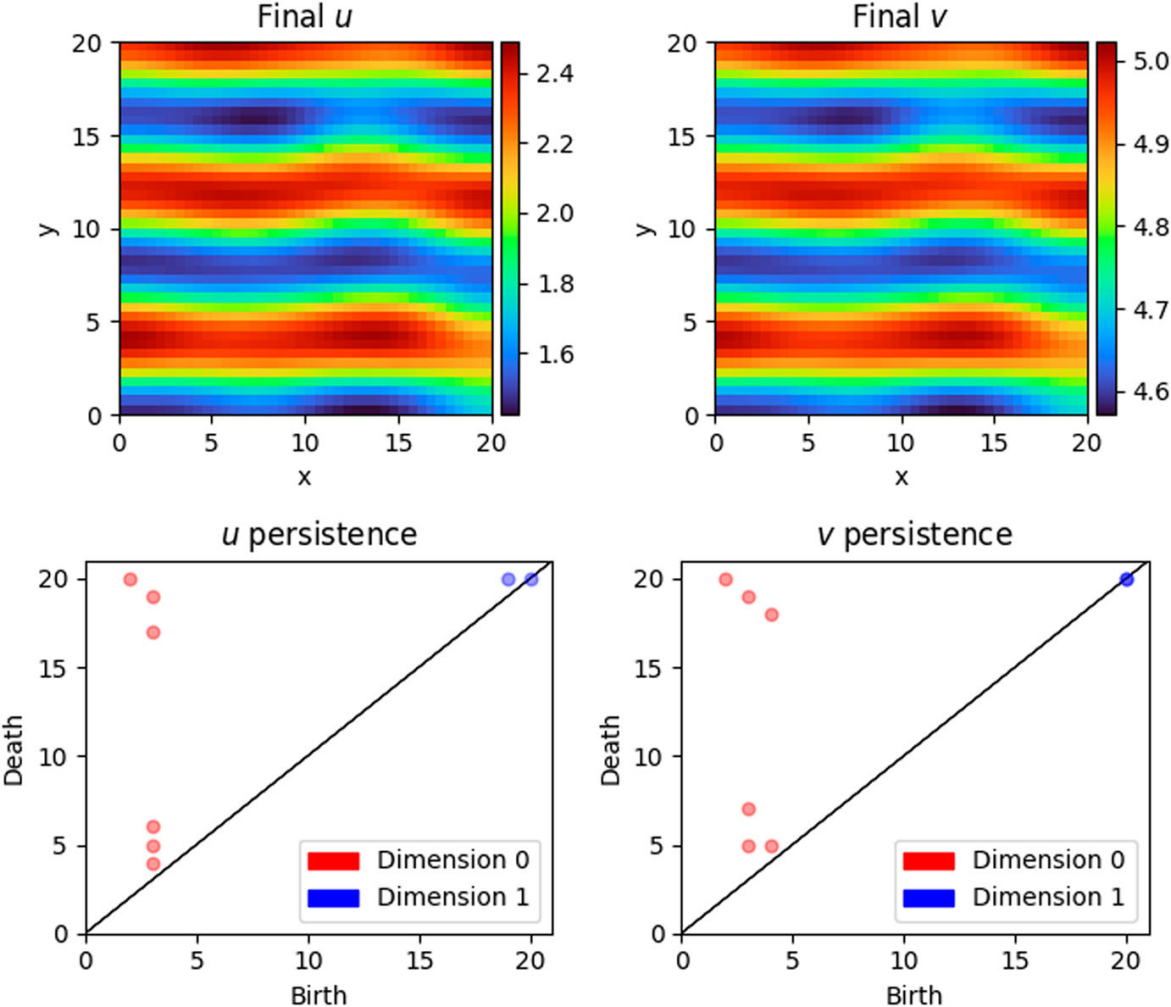
Another realisation of (CIMA), with persistence diagrams. As indicated by the red markers, representing persistent homology generators in degree 0, the sublevel sets of the solution surfaces are homeomorphic for a wide range of filtration values. Here $\alpha = 20$, $\beta = 1.35$, $\delta = 1.5$ and $\sigma = 11$

Similarly, Figure 5 shows typical realisations and persistence diagrams for a stripes pattern. In this case, both persistence diagram have three generators of homology in degree 0 that persist for many lower-star filtration values. In the bottom-left diagram, these represent the connected component associated to each blue stripe (minimum of *u*); in the bottom-right diagram, these represent the connected component associated to each red stripe (maximum of *v*).

Recalling the stability theorem from Subsection 3.2, we remark that perturbing the final concentration of *u* (respectively, *v*) by a small amount (in the supremum norm) also perturbs the persistence diagram associated with the final concentration of *u* (respectively, *v*) by a small amount (in Wasserstein distance). Since our numerical solutions will only ever be an approximation of the true solutions, this continuity is critical in demonstrating that the persistence diagrams, and the clustering that follows, are not sensitive to the small perturbations that are artefacts of numerical simulation.

### Remark 5.1

When the sublevel sets of the stable solutions (*u*, *v*) to a PDE such as (CIMA) are compact surfaces with boundary, we can conclude (via the classification of surfaces) that these isomorphisms on homology are induced by homeomorphisms of the solution manifold, so that the homeomorphism class of the manifold is, for a wide range of filtration values, stable to perturbations in the filtration value.

**Fig. 6:**
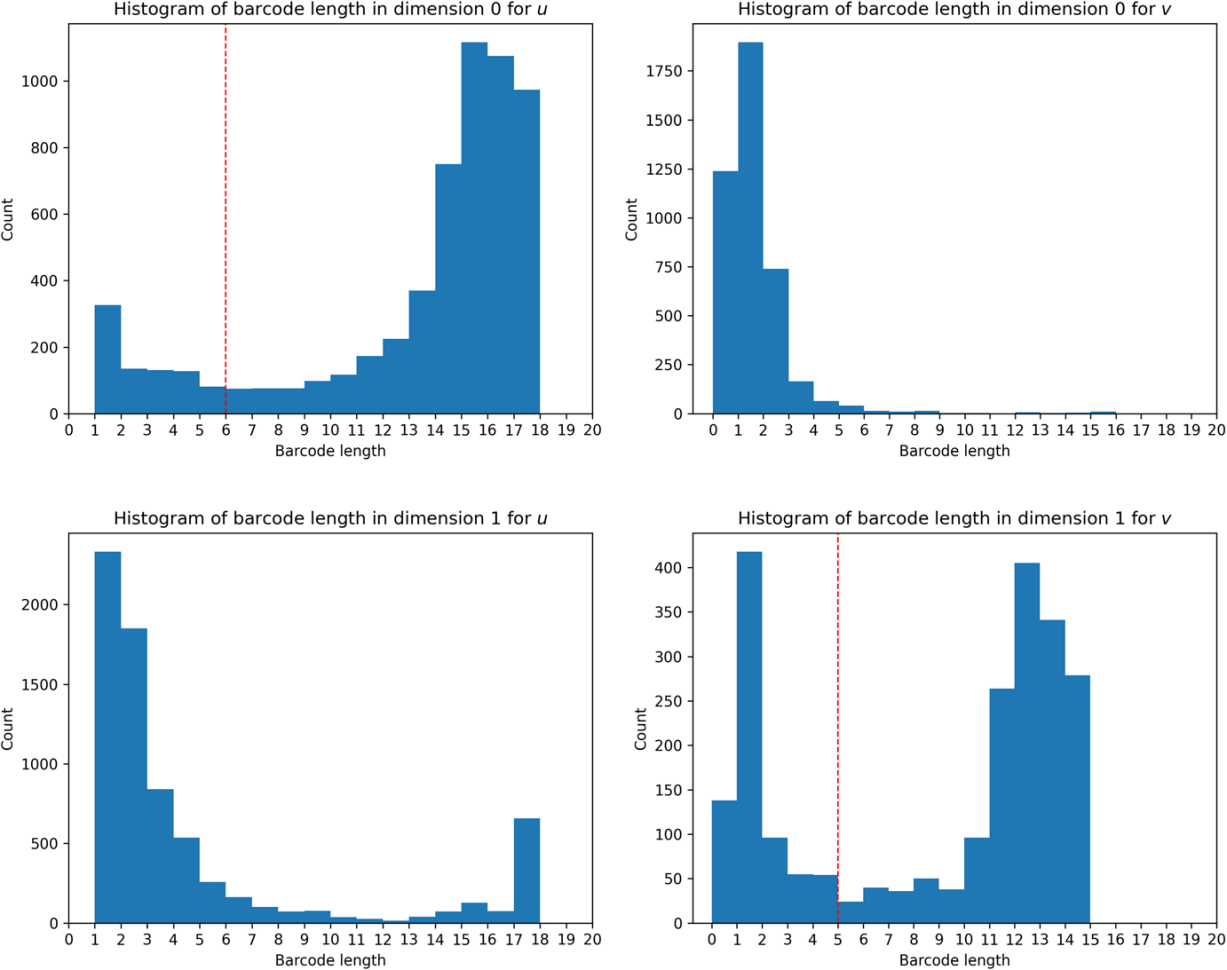
Histograms of barcode lengths of $B_j^u$ and $B_j^v$ with the proposed cutoffs indicated where applicable. Although there is multimodality in the lengths of $B_1^u$, the minimum lies at filtration value 11; taking a cutoff there would exclude some features that persist for many filtration values

One prominent feature of the figures is the existence of short barcodes, which capture short-lived topological features representing small perturbations in the manifold, which the stability theorems allow us to interpret as “noise". It is therefore natural to consider cutoffs for what lengths of barcodes are considered short enough to be attributed to such noise. We plot the density of barcode lengths for $B_j^u$ and $B_j^v$ and hope for multi-modality in the densities with a peak near 0, which would suggest a natural cutoff point at the minimum of the distribution between the two smallest dominant modes. As shown in Figure 6, this occurs for $B_0^u$, $B_1^u$ and $B_1^v$. Cutoffs are taken only for $B_0^u$ and $B_1^v$

When clustering, it is therefore possible to consider the *cleaned barcodes*, which are the barcodes with all short bars removed: these are all the bars in $B_1^u$, and all bars of length at least 5 in $B_0^u$, $B_0^v$ and $B_1^v$. In the setting of persistence diagrams, these bars correspond to points sufficiently far from the diagonal. The cleaned barcodes are denoted by $C_j^u$ and $C_j^v$ for $j=0,1$.

### Clustering in the CIMA and Schnakenberg Systems

For convenience, we refer to nodes whose pattern is spots (respectively stripes, labyrinths) as a *spots (respectively stripes, labyrinths) node*.

To plot figures that are more easily interpretable, each node is marked with the cluster it is assigned to, and clusters are colour-coded.

**Fig. 7:**
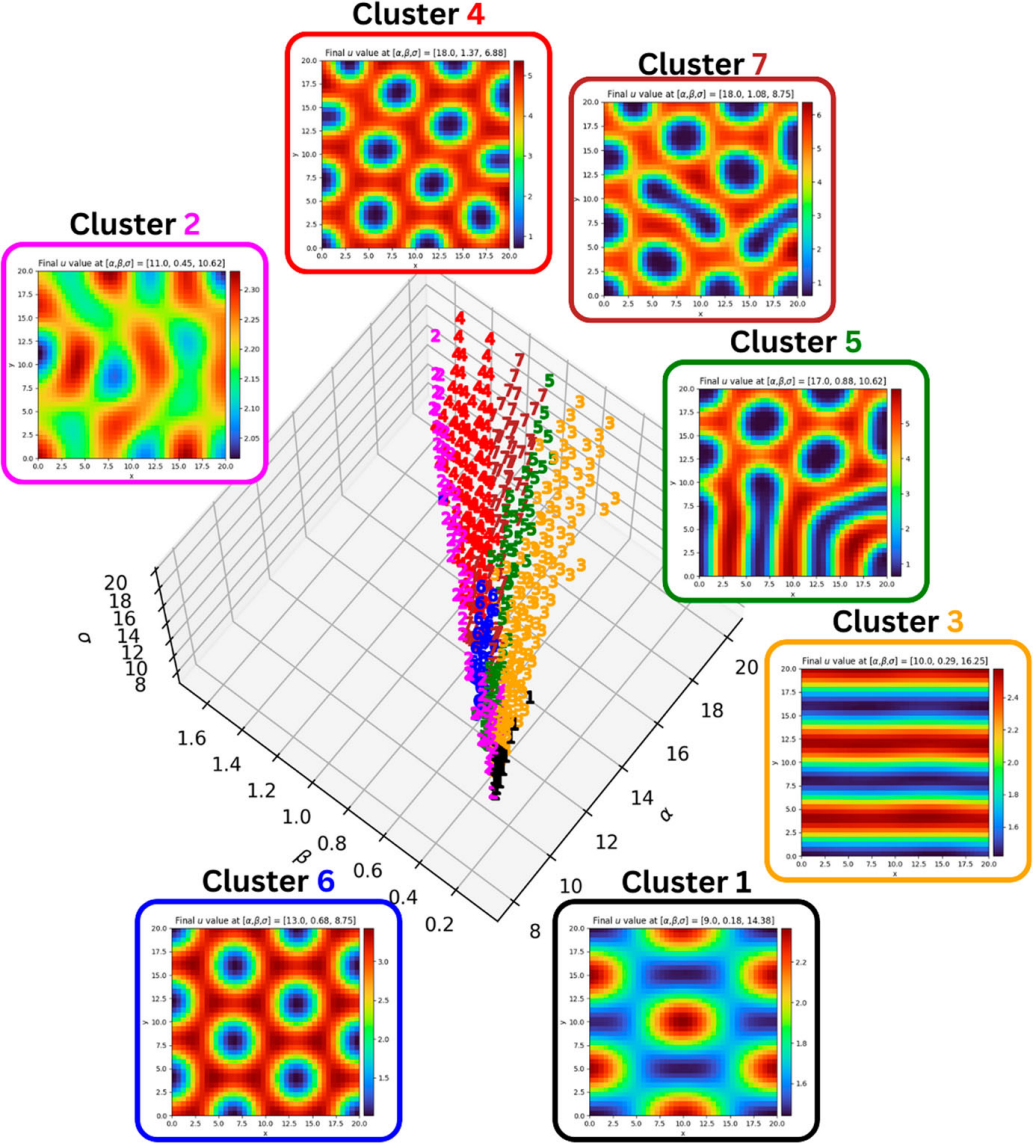
Topological clustering in the CIMA system (with $\delta = 1.5$) produced by the algorithm described in 4.3. Each point is coloured according to the cluster it is assigned to: cluster 1 has inverted spots; cluster 2 has broken stripes; cluster 3 has stripes; cluster 4 has spots; cluster 5 has stripy labyrinths; cluster 6 has spots; and cluster 7 has spotty labyrinths. with nodes in 6 displaying twelve spots, and nodes in 4 displaying thirteen or fourteen

For comparison, the methodology is slightly adapted and also applied to a well-studied Turing system based on chemical kinetics, namely the Schnakenberg model (Schnakenberg 1979), given by

S $$\begin{aligned} \begin{aligned} \frac{\partial u}{\partial \tau }&= \nabla ^2u + \alpha - u + u^2 v, \\ \frac{\partial v}{\partial \tau }&= \delta \nabla ^2v + \beta - u^2 v. \end{aligned} \end{aligned}$$

Unlike the (CIMA) system, the kinetics of the Schnakenberg system (S) are cross kinetics, in a sense that in a stable pattern, the densities of *u* and *v* are not aligned, as can be seen by examining the kinetic terms $\pm u^2 v$. Denoting the simplicial complex upon whose realisation we solve (S) by *L*, we therefore expect the homology groups $\operatorname {H}_k \left( \mathcal {U}_j L \right)$ and $\operatorname {H}_k \left( \mathcal {V}_j L \right)$ at intermediate filtration values *j* to be similar. We therefore adapt our choice of filtration, taking upper-star filtrations for both *u* and *v*. These filtrations are given by

$$\begin{aligned} \mathcal {U}_j L&= \left\{ \sigma \in L :u(|\tau |) \ge {\max u - \frac{\max u - \min u}{20}j} \; \text {for each 0-simplex } \tau \; \text {of } \sigma \right\} , \\ \mathcal {V}_j L&= \left\{ \sigma \in L :v(|\tau |) \ge {\max v - \frac{\max v - \min v}{20}j} \; \text {for each 0-simplex } \tau \; \text {of } \sigma \right\} . \end{aligned}$$

**Fig. 8:**
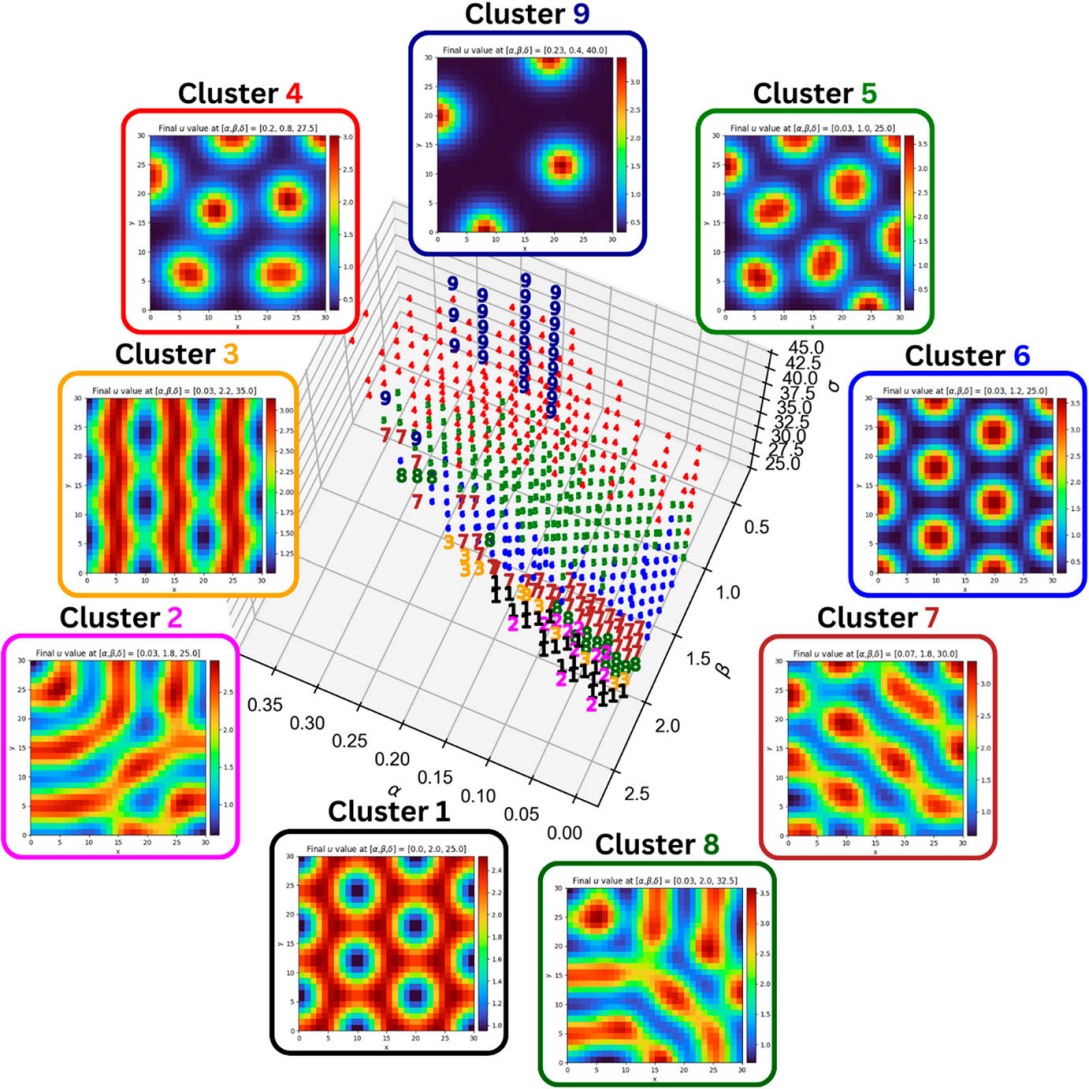
Topological clustering in the Schnakenberg system produced by the algorithm described in 4.3. Each point is coloured according to the cluster it is assigned to: cluster 1 has inverted spots; cluster 2 consists of messy stripes; cluster 3 has stripes; nodes in clusters 4, 5 and 6 have spots, with the number of spots increasing from one to the next; clusters 7 and 8 have broken stripes; finally, cluster 9 has very few (three to five) spots

By bounding the diffusion coefficient $\delta$ in the Schnakenberg system (S) system above and below yields a bounded Turing space. We fix $\delta \in [25,45]$ and obtain the clustering shown by Figure 8.

### Interpretation of Results

We now turn to the interpretation of our results from Section 5.

As shown by Figures 7 and 8, the clustering produced by our algorithm captures some of the diversity of stable patterns that can be produced by the CIMA and Schnakenberg systems, and in both cases shows a partitioning between the regions of the Turing space that exhibit each pattern.

**Fig. 9:**
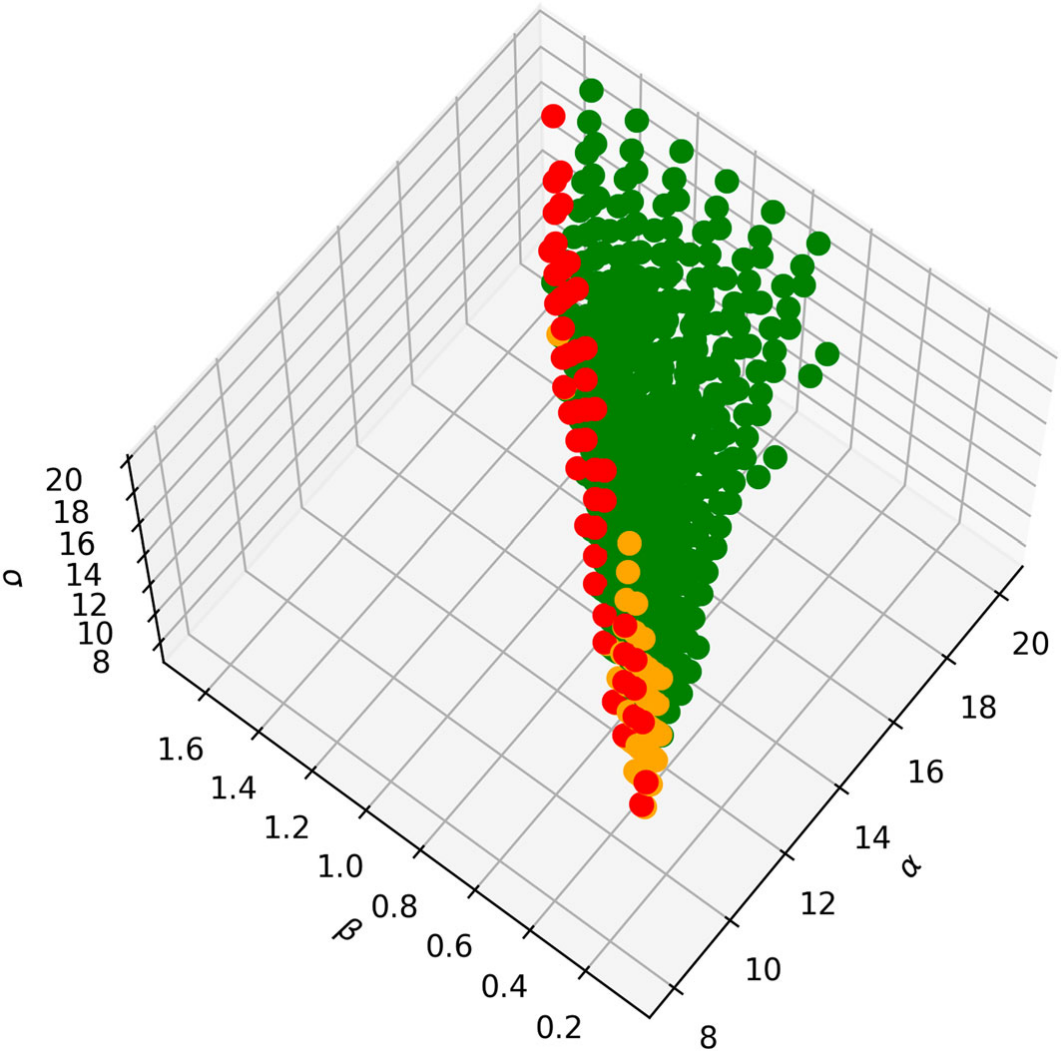
Difference between $\max u$ and $\min u$ for nodes in the Turing space of the CIMA system. Colouring is done according to which percentile the value of $\max u - \min u$ at a node: a node is coloured red if it is in the bottom $10\%$; a node that is not coloured red is coloured orange if it is in the bottom $25\%$; and all other nodes are coloured green. The Turing space is discretised as in Subsection 4.2

In the CIMA system, the clustering in figure 7 shows multiple features of interest. First it show a clear distinction between the stripes region (cluster 3) of the Turing space and the spotty regions (clusters 4 and 6), with the intermediate regions (clusters 5 and 7) showing labyrinthine patterns, indicating a gradual transition from stripes to spots. The second feature of interest is the deterioration of the pattern near the (C4) boundary of the Turing space, where we see the vast majority of cluster 2’s nodes. As expected, nodes near the (C4) boundary have the lowest difference between the maximum and minimum of *u* (See Figure 9).

In the Schnakenberg system, the clustering in Figure 8 suggests that, within the restricted Turing space, $\beta$ is the primary variable that determines the pattern type. For example, at the lowest values of $\beta$, we see nodes in clusters 9 (three to five spots) and 4 (six to eight spots) have few spots; increasing $\beta$ gives nodes in clusters 5 (nine to eleven spots) and 6 (twelve to thirteen spots); at high values of $\beta$, going from nodes in clusters 7 to 8 to 3 and 2, we observe stripes which become increasingly defined; right on the boundary of the Turing space, where the value of $\beta$ is maximal, the nodes in cluster 1 have inverted spots.

What is observed in both systems is that the topological clustering captures the parameter dependence of the systems well, and indicates that, away from the boundaries of the Turing space, the patterns are locally continuously dependent on the parameters. This confirms that, given bounds on the parameters, topological data analysis can be used to narrow down the subset of parameters a given observed pattern can lie in, unlocking potential paths towards parameter estimation and model selection, as detailed in Section 6.

The cautious reader may also be wary of the choice of the metric *d* in equation (5) on the Turing space. To address this, we also compared the performance of a couple of different metrics by comparing the clustering figures and their corresponding silhouette scores and found that all the metrics tested give comparable results both in terms of the clustering, and in terms of the silhouette scores. The metrics considered were given by

$$\begin{aligned} d_{1}\left( \theta _1, \theta _2\right)&{:}{=}\sum _{\begin{array}{c} j \in \{0,1\} \\ w \in \{u,v\} \end{array}} W_{2,2}\left( B_j^w(\theta _1) ,B_j^w(\theta _2)\right) ,\\ d_{2}\left( \theta _1, \theta _2\right)&{:}{=}\sum _{\begin{array}{c} j \in \{0,1\} \\ w \in \{u,v\} \end{array}} \sqrt{W_{2,2}\left( B_j^w(\theta _1) ,B_j^w(\theta _2)\right) ^2},\\ d_{\infty }\left( \theta _1, \theta _2\right)&{:}{=}\max _{\begin{array}{c} j \in \{0,1\} \\ w \in \{u,v\} \end{array}} W_{2,2}\left( B_j^w(\theta _1) ,B_j^w(\theta _2)\right) , \end{aligned}$$

noting that the metric in equation (5) is $d = d_1$.

Furthermore, the choice of metric may affect the precise number of spots or stripes in a cluster. We recall that the primary purpose of the (unsupervised) clustering is *not* to refine the clusters to an exact number of features, but rather classify the overall pattern topology in the final concentration of reactants.

A more general remark would be that, perhaps surprisingly, the vectorisation of the barcode data that we have provided (which is the most straightforward one) together with the Wasserstein distance, was sufficient to obtain well-defined clusters. It would be of interest to investigate the insights provided by clustering data obtained from other vectorisations, such as some of the ones discussed in Ali et al. (2023), though this is outside the scope of the paper.

Furthermore, we can investigate what the partitioning of the Turing space can inform us about the suitability of a model given observations of a pattern in a small neighbourhood of the Turing space. In the CIMA system, we see the “spots" clusters (6 and 7) both neighbour a labyrinthine region (cluster 5) of the Turing space, whereas in the Schnakenberg system, all bifurcations where the pattern changes from spots to labyrinths or stripes occur on the boundary of the spots clusters 5 and 6, with cluster 4 (whose nodes also have spots) only neighbouring other spots regions (5 and 9).

#### Remark 5.2

Another differentiator is the location of the Hopf bifurcation associated to the stability of the steady state in the absence of diffusion: in the CIMA system, the Hopf bifurcation occurs at the boundary adjacent to clusters 1 and 4 (inverted spots and stripes); in the Schnakenberg system, the Hopf bifurcation occurs at the boundary adjacent to clusters 4 and 9 (both spots).

In practice, one may be tempted to observe these phenomena by having parameters that traverse a path in the Turing space on a timescale much slower than that of the reaction and diffusion terms, noting the parameter timescale evolution has to be very slow to avoid the non-autonomy altering the pattern formation (Madzvamuse et al. 2010). However, the non-autonomy also removes the random initial condition whose role is to excite all the Fourier modes, replacing it with the initial condition of the previous parameter values’ stable pattern. This may lead to results becoming strongly dependent on the initial parameter values due to the sensitivity of mode selection.

## Discussion

In this section, we interpret the results of the previous section, discuss some of the limitations of the methodology, point out directions leading to potential improvements to the work, and suggest a few avenues of further research.

We begin by recalling the two objectives we first set out to investigate: our primary goal was to explore the feasibility of using topological data analysis to classify the topology of solutions to reaction diffusion systems; our secondary goal was discussing to what extent our findings could be applied to yield new insights into Turing systems, particularly into parameter estimation. We see that our topological approach is capable of partitioning the Turing space according to the persistent homology of the patterns for both pure (CIMA) and cross (Schnakenberg) kinetics in two-reactant systems, and find that the clusters can determine the type of pattern, and how the patterns relate to the parameters of the model.

Our results in Figures 7 and 8 show that the Turing space is partitioned into regions with topologically distinct solution manifolds, albeit with fuzzy boundaries. This is further supported by experiments which highlight that the clustering is not absolutely precise for nodes on the boundaries of a cluster (these are primarily nodes where solutions have labyrinths), and that the classification and its performance depends on choices such as the metric and the linkage method. Nevertheless, these minor differences do not refute our primary result that topological summaries are sufficient to capture the parameter dependence of patterns in the reaction diffusion systems considered.

Importantly, we observe that our framework can be used in any setting where spatially heterogeneous data is collected, either from simulations or experiments. Implementing this framework in such settings can therefore help analyse the system by exploring the parameter-dependence of the spatial patterns it produces.

When comparing the results for the CIMA system (pure kinetics) and the Schnakenberg system (cross kinetics), we observe that the choice of filtration plays a crucial role for two reasons: first, the choice of a lower- or upper-star filtration for the first species *u* allows us to apply our pipeline for both types of kinetics; second, the rescaling in the filtration allows a direct comparison of the persistent homology at different nodes, despite differences in pattern amplitude. In particular, the filtrations used throughout are defined in terms of the minimum and maximum values of the functions $u,v :|K| \rightarrow \mathbb {R}$, which enables direct comparison of patterns arising from the PDEs, though some nodes’ stable pattern will have very little spread in the concentrations of reactants, which may require careful interpretation.

Having discussed the clustering algorithm and its results, we are interested in other insights clustering can provide. We suggest the possibility of restricting the parameter space to one where the observations are topologically consistent using our clustering, so that we can restrict prior information for Bayesian parameter estimation and for model selection, but note that an explicit implementation is outside the scope of the paper. Ideally, the noise in the initial condition would be propagated to the clustering of final solutions of the PDEs: clustering should be carried out for a large number of different initial conditions (random perturbations of the steady state $(u_s, v_s)$) and the probability of exhibiting a particular pattern (as determined by the topological clustering) should be estimated at each point in the discretised parameter space. This empirical distribution of clusters could then be encoded as an informative prior for Bayesian inference (see (Campillo-Funollet et al. 2019), for example).

We also highlight the dependence of the results on a choice of model: the topological summaries obtained from the parameter sweep may be highly dependent on the specific form of the kinetics chosen for the model. This becomes extremely relevant in a biological setting, where the modeller often has little information about the kinetics beyond observing whether they are pure or cross kinetics. For purposes of parameter inference, it is therefore crucial that the kinetics are known with a degree of certainty (for example, that obtained by analysing chemical systems, where the reaction terms are highly restricted).

This comes with an interesting dual when used for the purposes of model selection when extensive observational data is available. It is known that when carrying out model selection, we require more than static observations: Woolley et al. have shown that a non-unique Turing system can be explicitly constructed to display a chosen pattern (spots or stripes/labyrinths) within any specified region of the Turing space (Woolley et al. 2021). The topological clustering can nevertheless be used when selecting between a fixed set of candidate models by considering (sufficiently large) regions of the Turing space, asking how the clusters partition these regions, and comparing this partition to data, and to analytically known bifurcations of the models (such as the Hopf bifurcation, see for example the comparison in Remark 5.2). This procedure allows us to add topological clustering to the broad existing toolkit for model selection that is used in the study of spatially heterogeneous models.

Another avenue for further research is in investigating how persistent homology can be used in the case of spatially heterogeneous parameters (Krause et al. 2020; Van Gorder 2021). The upshot of Remark 5.1 is that in a chemical or biological system wherein a pattern is determined by positional information (e.g. thresholds of the reactants’ densities, see Wolpert (1969)), the topology of the pattern is stable to perturbations in the threshold. If we allow ourselves to assume that a model with spatially homogeneous parameters is appropriate, combining this with the stability provided by Theorem 3.15, we can interpret this stability as evidence of the topological robustness of patterns arising via a combination of positional information and reaction diffusion mechanisms (Green and Sharpe 2015).

In summary, by using the persistent homology of solution manifolds to reaction diffusion systems with respect to lower- and upper-star filtrations, we have developed a pipeline that uses the 2-Wasserstein distance to cluster points in the Turing space according to the type of pattern that emerges from a random initial condition. Using the results of applying this algorithm to the CIMA and Schnakenberg systems, we demonstrate this clustering partitions the Turing space into topologically distinct patterns and suggest ways our pipeline can be incorporated into existing toolkits for the analysis of spatially heterogeneous patterns arising from reaction diffusion models.

## Data Availability

All codes and generated data used in this paper are available on GitHub.

## References

[CR1] Ali D, Asaad A, Jimenez M, Nanda V, Paluzo-Hidalgo E, Soriano-Trigueros M (2023) A survey of vectorization methods in topological data analysis. IEEE Trans Pattern Anal Mach Intell 45(12):14069–14080. 10.1109/TPAMI.2023.3308391 37647183 10.1109/TPAMI.2023.3308391

[CR2] Arcuri P, Murray JD (1986) Pattern sensitivity to boundary and initial conditions in reaction-diffusion models. J Math Biology 24(2):141–165. 10.1007/BF00275996 3755746

[CR3] Boissonnat J, Dey TK, Maria C (2013) The compressed annotation matrix: an efficient data structure for computing persistent cohomology. In Hans L. Bodlaender and Giuseppe F. Italiano, editors, Algorithms – ESA 2013, pages 695–706, Berlin, Heidelberg, Springer. 10.1007/978-3-642-40450-4_59

[CR4] Carlsson G (2009) Topology and data. Bull Amer Math Soc 46:255–308. 10.1090/S0273-0979-09-01249-X

[CR5] Castets V, Dulos E, Boissonade J, de Kepper P (1990) Experimental evidence of a sustained standing Turing-type nonequilibrium chemical pattern. Phys Rev Lett 64(24):2953–2956. 10.1103/PhysRevLett.64.2953 10041855 10.1103/PhysRevLett.64.2953

[CR6] Campillo-Funollet E, Venkataraman C, Madzvamuse A (2019) Bayesian parameter identification for Turing systems on stationary and evolving domains. Bull Math Biol 81(1):81–104. 10.1007/s11538-018-0518-z 30311137 10.1007/s11538-018-0518-z PMC6320356

[CR7] Cohen-Steiner D, Edelsbrunner H, Harer J (2007) Stability of persistence diagrams. Discrete Comput Geom 37(1):103–120. 10.1007/s00454-006-1276-5

[CR8] Cohen-Steiner D, Edelsbrunner H, Harer J, Mileyko Y (2010) Lipschitz Functions Have Lp-Stable Persistence. Found Comput Math 10(2):127–139. 10.1007/s10208-010-9060-6

[CR9] Dey TK, Fan F, Wang Y (2014) Computing topological persistence for simplicial maps. In Proceedings of the thirtieth annual symposium on Computational geometry, SOCG’14, pages 345–354, New York, NY, USA. Association for Computing Machinery. 10.1145/2582112.2582165

[CR10] Dillon R, Maini PK, Othmer HG (1994) Pattern formation in generalized Turing systems. J Math Biol 32(4):345–393. 10.1007/BF00160165

[CR11] de Silva V, Morozov D, Vejdemo-Johansson M (2011) Dualities in persistent (co)homology. Inverse Prob 27(12):124003. 10.1088/0266-5611/27/12/124003

[CR12] de Silva V, Morozov D, Vejdemo-Johansson M (2011) Persistent cohomology and circular coordinates. Discrete Comput Geom 45(4):737–759. 10.1007/s00454-011-9344-x

[CR13] Edelsbrunner H, Harer J (2010) Computational Topology: An Introduction. American Mathematical Society. 10.1007/978-3-540-33259-6_7

[CR14] Economou AD, Ohazama A, Porntaveetus T, Sharpe PT, Kondo S, Basson MA, Gritli-Linde A, Cobourne MT, Green JBA (2012) Periodic stripe formation by a Turing mechanism operating at growth zones in the mammalian palate. Nat Genet 44(3):348–351 Publisher: Nature Publishing Group. 10.1038/ng.1090 10.1038/ng.1090 PMC3303118 22344222

[CR15] Ermentrout B (1991) Stripes or spots? Nonlinear effects in bifurcation of reaction—diffusion equations on the square. Proceedings of the Royal Society of London. Series A: Mathematical and Physical Sciences, 434(1891):413–417, Publisher: Royal Society. 10.1098/rspa.1991.0100

[CR16] Ge Z (2023) The hidden order of Turing patterns in arid and semi-arid vegetation ecosystems. Proceedings of the National Academy of Sciences, 120(42):e2306514120, Publisher: Proceedings of the National Academy of Sciences. 10.1073/pnas.2306514120 10.1073/pnas.2306514120 PMC10589663 37816060

[CR17] Ghrist R (2014) Elementary Applied Topology. Self-published, first edition, https://www2.math.upenn.edu/~ghrist/notes.html

[CR18] Gierer A, Meinhardt H (1972) A theory of biological pattern formation. Kybernetik 12(1):30–39. 10.1007/BF00289234 4663624 10.1007/BF00289234

[CR19] Gaspar V, Showalter K (1990) Simple model for the oscillatory iodate oxidation of sulfite and ferrocyanide. J. Phys. Chem., 94(12):4973–4979, Publisher: American Chemical Society. 10.1021/j100375a040

[CR20] Green JBA, Sharpe J (2015) Positional information and reaction-diffusion: two big ideas in developmental biology combine. Development 142(7):1203–1211. 10.1242/dev.114991 25804733 10.1242/dev.114991

[CR21] Glover JD, Sudderick ZR, Shih BB, Batho-Samblas C, Charlton L, Krause AL, Anderson C, Riddell J, Balic A, Li J, Klika V, Woolley TE, Gaffney EA, Corsinotti A, Anderson RA, Johnston LJ, Brown SJ, Wang S, Chen Y, Crichton ML, Headon DJ (2023) The developmental basis of fingerprint pattern formation and variation. Cell 186(5):940-956.e20. 10.1016/j.cell.2023.01.015 36764291 10.1016/j.cell.2023.01.015

[CR22] Hatcher A (2002) Algebraic Topology. Cambridge University Press, https://pi.math.cornell.edu/~hatcher/AT/ATpage.html

[CR23] Krause AL, Gaffney EA, Maini PK, Klika V (2021) Modern perspectives on near-equilibrium analysis of Turing systems. Philosophical Transactions of the Royal Society A: Mathematical, Physical and Engineering Sciences 379(2213):20200268, Publisher: Royal Society. 10.1098/rsta.2020.0268 10.1098/rsta.2020.0268 PMC8580451 34743603

[CR24] Krause AL, Klika V, Woolley TE, Gaffney EA (2020) From one pattern into another: analysis of Turing patterns in heterogeneous domains via WKBJ. Journal of The Royal Society Interface 17(162):20190621, Publisher: Royal Society. 10.1098/rsif.2019.0621 10.1098/rsif.2019.0621 PMC7014807 31937231

[CR25] Kuznetsov YA (2004) Elements of Applied Bifurcation Theory, volume 112 of Applied Mathematical Sciences. Springer, New York, NY, 10.1007/978-1-4757-3978-7

[CR26] Lengyel I, Epstein IR (1991) Modeling of Turing structures in the chlorite-iodide-malonic acid-starch reaction system. Science 251(4994):650–652, Publisher: American Association for the Advancement of Science. 10.1126/science.251.4994.650 10.1126/science.251.4994.650 17741380

[CR27] Lengyel I, Epstein IR (1992) A chemical approach to designing Turing patterns in reaction-diffusion systems. Proceedings of the National Academy of Sciences 89(9):3977–3979, Publisher: Proceedings of the National Academy of Sciences. 10.1073/pnas.89.9.3977 10.1073/pnas.89.9.3977 PMC525614 11607288

[CR28] Madzvamuse A, Gaffney EA, Maini PK (2010) Stability analysis of non-autonomous reaction-diffusion systems: the effects of growing domains. J Math Biol 61(1):133–164. 10.1007/s00285-009-0293-4 19727733 10.1007/s00285-009-0293-4

[CR29] Meinhardt H, Klingler M (1987) A model for pattern formation on the shells of molluscs. J Theor Biol 126(1):63–89. 10.1016/S0022-5193(87)80101-7

[CR30] Mileyko Y, Mukherjee S, Harer J (2011) Probability measures on the space of persistence diagrams. Inverse Problems - INVERSE PROBL, 27, 10.1088/0266-5611/27/12/124007

[CR31] McDonald RA, Neuhausler R, Robinson M, Larsen LG, Harrington HA, Bruna M (2023) Zigzag persistence for coral reef resilience using a stochastic spatial model. Journal of The Royal Society Interface 20(205):20230280, Publisher: Royal Society. 10.1098/rsif.2023.0280 10.1098/rsif.2023.0280 PMC10445017 37608713

[CR32] Müller P, Rogers KW, Jordan BM, Lee JS, Robson D, Ramanathan S, Schier AF (2012) Differential diffusivity of nodal and lefty underlies a reaction-diffusion patterning system. Science 336(6082):721–724, Publisher: American Association for the Advancement of Science. 10.1126/science.1221920 10.1126/science.1221920 PMC3525670 22499809

[CR33] Murray JD (2003) Mathematical Biology: II: Spatial Models and Biomedical Applications, volume 18 of Interdisciplinary Applied Mathematics. Springer, New York, NY, 10.1007/b98869

[CR34] McGuirl MR, Volkening A, Sandstede B (2020) Topological data analysis of zebrafish patterns. Proc Natl Acad Sci 117(10):5113–5124. 10.1073/pnas.1917763117 32098851 10.1073/pnas.1917763117 PMC7071871

[CR35] Nanda V (2024) Computational Algebraic Topology Lecture Notes. Available at https://people.maths.ox.ac.uk/nanda/cat/TDANotes.pdf

[CR36] Nardini JT, Stolz BJ, Flores KB, Harrington HA, Byrne HM (2021) Topological data analysis distinguishes parameter regimes in the Anderson-Chaplain model of angiogenesis. PLOS Computational Biology 17(6):e1009094, Publisher: Public Library of Science. 10.1371/journal.pcbi.1009094 10.1371/journal.pcbi.1009094 PMC8270459 34181657

[CR37] Otter N, Porter MA, Tillmann U, Grindrod P, Harrington HA (2017) A roadmap for the computation of persistent homology. EPJ Data Sci 6(1):17. 10.1140/epjds/s13688-017-0109-5 32025466 10.1140/epjds/s13688-017-0109-5 PMC6979512

[CR38] Pedregosa F, Varoquaux G, Gramfort A, Michel V, Thirion B, Grisel O, Blondel M, Prettenhofer P, Weiss R, Dubourg V, Vanderplas J, Passos A, Cournapeau D, Brucher M, Perrot M, Duchesnay E (2011) Scikit-learn: Machine learning in Python. Journal of Machine Learning Research 12:2825–2830 (https://dl.acm.org/doi/10.5555/1953048.2078195)

[CR39] Rousseeuw PJ (1987) Silhouettes: A graphical aid to the interpretation and validation of cluster analysis. J Comput Appl Math 20:53–65. 10.1016/0377-0427(87)90125-7

[CR40] Schnakenberg J (1979) Simple chemical reaction systems with limit cycle behaviour. J Theor Biol 81(3):389–400. 10.1016/0022-5193(79)90042-0 537379 10.1016/0022-5193(79)90042-0

[CR41] Schenck H (2022) Algebraic Foundations for Applied Topology and Data Analysis. Mathematics of data Springer International Publishing. 10.1007/978-3-031-06664-1

[CR42] Stolz BJ, Dhesi J, Bull JA, Harrington HA, Byrne HM, Yoon IHR (2024) Relational persistent homology for multispecies data with application to the tumor microenvironment. Bull. Math. Bio. 86(11):128. 10.1007/s11538-024-01353-6 39287883 10.1007/s11538-024-01353-6 PMC11408586

[CR43] Stolz BJ, Kaeppler J, Markelc B, Braun F, Lipsmeier F, Muschel RJ, Byrne HM, Harrington HA (2022) Multiscale topology characterizes dynamic tumor vascular networks. Science Advances, 8(23), 10.1126/sciadv.abm2456 10.1126/sciadv.abm2456 PMC9187234 35687679

[CR44] The GUDHI Project. (2021) GUDHI User and Reference Manual. GUDHI Editorial Board, 3.4.1 edition, https://gudhi.inria.fr/doc/3.4.1/

[CR45] The editors (2022) Turing patterns, 70 years later. Nat. Comput. Sci., 2(8):463–464, Publisher: Nature Publishing Group. 10.1038/s43588-022-00306-0 10.1038/s43588-022-00306-0 38177795

[CR46] Turing AM (1952) The Chemical Basis of Morphogenesis. Philosophical Transactions of the Royal Society of London. Series B, Biological Sciences, 237(641):37–72, Publisher: The Royal Society. 10.1007/BF02459572

[CR47] Topaz CM, Ziegelmeier L, Halverson T (2015) Topological data analysis of biological aggregation models. PLOS ONE 10(5):e0126383, Publisher: Public Library of Science. 10.1371/journal.pone.0126383 10.1371/journal.pone.0126383 PMC4430537 25970184

[CR48] Van Gorder RA (2021) Pattern formation from spatially heterogeneous reaction-diffusion systems. Philosophical Transactions of the Royal Society A: Mathematical, Physical and Engineering Sciences 379(2213):20210001,Publisher: Royal Society. 10.1098/rsta.2021.0001 10.1098/rsta.2021.0001 34743604

[CR49] Virtanen P, Gommers R, Oliphant TE, Haberland M, Reddy T, Cournapeau D, Burovski E, Peterson P, Weckesser W, Bright J, van der Walt SJ, Brett M, Wilson J, Millman KJ, Mayorov N, Nelson ARJ, Jones E, Kern R, Larson E, Carey CJ, Polat İ, Feng Y, Moore EW, VanderPlas J, Laxalde D, Perktold J, Cimrman R, Henriksen I, Quinteo EA, Harris CR, Archibald AM, Ribeiro AH, Pedregosa F, van Mulbregt P, and SciPy 1.0 Contributors (2020) SciPy 1.0: Fundamental Algorithms for Scientific Computing in Python , Nat Methods 17:261–272. 10.1038/s41592-019-0686-2 10.1038/s41592-019-0686-2 PMC7056644 32015543

[CR50] Volkening A (2024) Methods for quantifying self-organization in biology: a forward-looking survey and tutorial, arXiv:2407.10832 [q-bio]

[CR51] Wasserman L (2018) Topological data analysis. Annual Review of Statistics and Its Application, 5(Volume 5, 2018):501–532, Publisher: Annual Reviews. 10.1146/annurev-statistics-031017-100045

[CR52] Woolley TE, Krause AL, Gaffney EA (2021) Bespoke Turing Systems. Bull Math Biol 83(5):41. 10.1007/s11538-021-00870-y 33740210 10.1007/s11538-021-00870-y PMC7979634

[CR53] Wolpert L (1969) Positional information and the spatial pattern of cellular differentiation. J Theor Biol 25(1):1–47. 10.1016/S0022-5193(69)80016-0 4390734 10.1016/s0022-5193(69)80016-0

[CR54] Yang J, Fang H, Dhesi J, Yoon IH, Bull JA, Byrne HM, Harrington HA, Grindstaff G (2025) Topological classification of tumour-immune interactions and dynamics. J Math Biol 91(25). 10.1007/s00285-025-02253-6 PMC12325540 40762719

[CR55] Zomorodian A, Carlsson G (2005) Computing Persistent Homology. Discrete Comput Geom 33(2):249–274. 10.1007/s00454-004-1146-y

[CR56] Zwicker D (2020) py-pde: A Python package for solving partial differential equations. Journal of Open Source Software 5(48):2158. 10.21105/joss.02158

